# Ameliorative Effects of Aerobic Exercise Combined With *Lycium barbarum* Polysaccharide‐Mediated Gut Microbiota Remodeling on Glycolipid Abnormalities in Type 2 Diabetic Rats

**DOI:** 10.1002/fsn3.71503

**Published:** 2026-02-01

**Authors:** Jing‐feng Wang, Shuo Feng, Xuan Cao, Xiao‐lin Li

**Affiliations:** ^1^ Harbin Sport University City Harbin Heilongjiang Province China

**Keywords:** aerobic exercise, gut microbiota, *Lycium barbarum* polysaccharide, short‐chain fatty acids, type 2 diabetes

## Abstract

Exercise plays an important role in improving type 2 diabetes (T2DM) by regulating systemic metabolism and enhancing glycemic control. *Lycium barbarum* polysaccharide (LBP), a natural bioactive component, also exhibits potential for lowering blood glucose and alleviating diabetes‐related symptoms. However, the underlying mechanisms by which exercise, LBP, or their combination alleviate T2DM remain largely unclear from the perspective of gut microbiota. In this study, we used 16S rDNA sequencing to analyze gut microbiota, aiming to investigate the roles of aerobic exercise and LBP in T2DM and explore their molecular mechanisms. We found that both aerobic exercise and LBP could modulate gut microbiota—promoting the proliferation of beneficial bacteria, reducing harmful bacteria, and optimizing intestinal microecology—while regulating gut microbial composition and metabolism to inhibit inflammatory responses. Additionally, they improved gut microbiota homeostasis to enhance cellular insulin sensitivity, optimize glucose catabolism and metabolism, regulate lipid metabolism, and reduce abnormal blood lipid accumulation. Notably, aerobic exercise combined with LBP exerted a more significant effect on gut microbiota modulation, thereby yielding better therapeutic outcomes for T2DM. Mechanistically, the regulation of gut microbiota by aerobic exercise and LBP in T2DM rats both involved the AMPK/PGC‐1α pathway, suggesting this may be a key link between gut microbiota and T2DM. Furthermore, isobutyric acid and its associated gut microbiota may play a critical role in the T2DM‐improving effects of aerobic exercise and LBP, warranting focused investigation in future studies.

## Graphical abstract

This schematic illustrates the protective effects of aerobic exercise and Lycium barbarum polysaccharide in a rat model of type 2 diabetes mellitus (T2DM), which was induced by high‐fat/high‐sugar feeding and streptozotocin (STZ) injection. Both interventions target the gut microbiota to promote short‐chain fatty acid production, thereby regulating energy metabolic pathways. This modulation ameliorates key T2DM‐related pathological processes—including inflammatory responses, dysregulated lipid/glucose metabolism, and insulin resistance—ultimately alleviating T2DM progression.

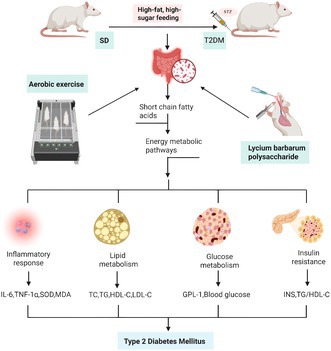

## 1 Introduction

Maintaining a balanced, reciprocal interaction between the host and intestinal microecology is crucial, as gut microbiota is a key regulator of host health. Many chronic diseases, including type 2 diabetes mellitus (T2DM)—a globally prevalent metabolic disorder—are linked to intestinal microecological dysbiosis; changes in gut microbial composition, a major environmental variable, further associate with metabolic impairments (Yehualashet and Yikna 2021). T2DM incidence and mortality continue to rise, posing a significant public health challenge. Currently incurable, its interventions primarily focus on glycemic control and managing complications, with gut microbiota playing a critical regulatory role in its development. External factors like dietary imbalance and physical inactivity have also driven a marked increase in T2DM incidence (Kang et al. 2022).

As a non‐pharmacological intervention, exercise prevents and improves T2DM by boosting energy expenditure, metabolic rate, glucose homeostasis, and insulin sensitivity (Balducci et al. 2014). It also enhances gut microbial biodiversity, beneficial bacterial abundance, and microbial metabolic capacity (Motiani et al. 2020; Li and Guo 2023), while reducing tumor necrosis factor levels in intestinal lymphocytes, stimulating antioxidant synthesis, and inhibiting inflammatory factor release (Campbell and Wisniewski 2017)—making it a key lifestyle strategy for improving health and reducing chronic disease risk (Keirns et al. 2020). Modulating gut microbiota via probiotics, prebiotics, synbiotics, or fecal microbial transplantation is another promising T2DM treatment (Iatcu et al. 2022). For instance, *Lycium barbarum* polysaccharide (LBP) exhibits prebiotic activity, balancing intestinal microbial composition, enhancing host bacterial abundance, and improving immunity (Zhu et al. 2020). Recent studies further highlight its health benefits, including antioxidant, anti‐inflammatory, anti‐tumor, neuroprotective, glucose‐lowering, and lipid‐lowering effects (Li et al. 2024).

Most existing T2DM studies focus on either aerobic exercise (e.g., its effects on metabolic indices) or LBP (e.g., its hypoglycemic efficacy), with few exploring their combination. This study is the first to use aerobic exercise combined with LBP as an intervention, investigating their individual and synergistic effects on T2DM from a gut microbiota perspective. This innovative combined intervention may yield more pronounced benefits than single interventions, potentially providing a more effective non‐pharmacological treatment option for T2DM patients.

## 2 Materials and Methods

### 2.1 Laboratory Animals and Reagents

Purchased from the Centre for Safety Assessment of Heilongjiang University of Traditional Chinese Medicine, 35 SPF‐grade 8‐week‐old male SD rats served as experimental animals in this work. LBP came from Shanghai Yuanye Biotechnology Co. LTD. Detailed experimental reagents and equipment are listed in Tables S1 and S2. The study was approved by the Academic Ethics Committee of Harbin Sport University (2024056).

### 2.2 Experimental Grouping and Animal Model Establishment

The 35 male SD rats were chosen; 6 of them were randomly chosen to create a blank control group (Control, 6 rats) following 1 week of adaptive eating on normal feed; the remaining 29 rats were T2DM modeled. Eight weeks of high‐sugar, high‐fat diet produced insulin resistance in the modeled rats. First fed high‐sugar and high‐fat chow (Feed ratio: 10% lard, 20% sucrose, 2.5% cholesterol, 0.5% sodium cholate, 67% maintenance feed) for 8 weeks to establish insulin resistance, the modeling rats were fasted without water for 12 h at the end of the feeding period. A single injection of 1% streptozotocin solution (35 mg/kg) was administered into the tail vein to simulate the onset of T2DM. The fasting blood glucose concentration of the rats was measured 72 h after the injection, and the modeling was considered successful if the concentration was ≥ 16.7 mmol/L. The final modeling was successful in 24 rats. Finally, 24 rats were successfully modeled. By means of a random grouping technique, the 24 T2DM rats were split into four groups: blank control group (T2DM group, 6), LBP intervention group (T2DM + LBP group, 6), exercise training group (T2DM + E group, 6), and exercise combined with LBP group (T2DM + LBP + E group, 6). Relative humidity of 55%–70%, a room temperature of 20°C–25°C, and a clean, orderly environment defined the well‐ventilated laboratory. The rats were housed in separate cages, 6 rats per cage, with 12 h of light exposure per day, and the rats were free to eat and drink.

### 2.3 Aerobic Exercise Intervention Program

Means of exercise intervention: Week 1 was the adaptive intervention phase. In order to allow the rats' bodies to gradually adapt to the running platform exercise and to avoid injuries or excessive fatigue caused by high‐intensity exercise at the beginning, the rats in the exercise group were acclimatized to the running platform at a speed of 10 m/min for 10 min, and then the running platform speed was incremented by 2 m/min each day, and the training time was increased by 10 min, until reaching a speed of 20 m/min for 60 min on the 6th day, and resting on the 7th day (Kleinert et al. 2018; Saraceni and Broderick 2007; Chiasera et al. 2000). The slope was set at 5° to simulate an environment closer to the actual exercise scenario and also to help exercise the leg muscle strength and balance of the rats (Lin et al. 2021). Weeks 2 through 13 were the formal training phase. In order to maintain and further improve the aerobic metabolic capacity, cardiovascular function, and muscular endurance of the rats, the present study entered the formal intervention phase at the speed, time, and incline of day 6 of the acclimatization training from week 2 until the end of the intervention cycle (Haram et al. 2009). In order to be able to ensure sufficient exercise stimulation to promote adaptive changes in various body systems and also to give the rats appropriate rest time to avoid fatigue and injury caused by overtraining, all rats in this study were trained once a day for 5 days per week (Zhang et al. 2023).

### 2.4 Dosage Information/Dosage Regimen

Administration was performed at 1 h after the rats' morning feeding daily. LBP was prepared as an aqueous solution at a concentration of 200 mg/(kg·bw·day) and was given 2 mL per dose by gavage. The gavage is carried out once a day for 12 weeks. Rationalization: 200 mg/(kg·bw·day) based on body weight, which can be administered accurately according to the individual differences of rats, and this dose can ensure that the drug can achieve certain pharmacological effects without producing serious toxic side effects due to overdose. The gastrointestinal tract of rats is active 1 h after feeding in the early morning. Fixed administration at this time is conducive to the maintenance of stable blood concentration and makes it easy to assess the efficacy of the drug. The use of distilled water as a solvent will not interfere with the drug and will help in accurate dispensing. The volume of 2 mL per gavage is suitable to ensure the distribution of the drug without increasing the gastrointestinal burden on the rats. The dosing arrangement of once daily for 12 weeks can maintain the sustained action of the drug and provide sufficient data for the study by observing the long‐term efficacy and mechanism of the drug. In addition, dosage of LBP could be supplemented either by regular diet (LBP) or by corresponding supplements. The human equivalent dose is calculated using the following formula: $\begin{array}{c} \mathrm{HED}\;\left(\text{human equivalent dose}\right)=\text{Animal dose}\left(\mathrm{mg}/\mathrm{kg}\right) \end{array}$ $\begin{array}{c} \times \frac{\text{Animal weight}\left(\mathrm{kg}\right)}{\text{Human weight}\left(\mathrm{kg}\right)}\times \frac{\text{Human body surface area}\;\left({\mathrm{ms}}^{²}\right)}{\text{Animal body surface area}\;\left({\mathrm{ms}}^{²}\right)} \end{array}$. The human equivalent dose is 44.3 mg/(kg·bw·day) (Ma et al. 2022; Liu et al. 2019, 2021; Ding et al. 2022; Lai et al. 2022).

### 2.5 General and Fasting Blood Sugar Test

Rats in every group had daily mental state and activity level observed. Every week, the weight of the rats was noted concurrently. For every group of rats, a fasting blood glucose test was performed simultaneously every week; the rats were fasted 12 h before the test. Rats' blood samples were taken by tail vein punctures, and test strips and a matching blood glucose detector were used to ascertain blood glucose content.

### 2.6 Enzyme‐Linked Immunosorbent Assay (ELISA)

Serum triglyceride (TG), total cholesterol (TC), high‐density lipoprotein (HDL‐C/LDL‐C), insulin (Ins), interleukin‐6 (IL‐6), tumor necrosis factor‐α (TNF‐α), glucagon peptide (GLP‐1), malondialdehyde (MDA), and superoxide dismutase (SOD) levels were detected by an ELISA kit matching the indexes. The rat serum samples were thoroughly mixed with 1 mL of PBS (pH 7.2–7.4). Next, set up a 96‐well plate by adding 50 μL of different standard concentrations into the standard wells, leaving the blank wells without samples or enzyme‐labeled reagent. For the test wells, dilute the samples by mixing 10 μL of serum with 40 μL of diluent—make sure to pipette this mixture to the bottom of the well without touching the sides, and mix it gently. Then, add 100 μL of enzyme‐labeled reagent to all wells except the blanks, cover the plate, and let it sit at 37°C for 60 min. At the same time, prepare a 20× wash buffer by mixing it 1:20 with distilled water for later use. After incubation, add 50 μL each of Developer A and B to all wells, mix gently, and incubate in the dark at 37°C for 15 min. Stop the reaction by adding 50 μL of stop solution, which will quickly change the color from blue to yellow, and then measure the absorbance (OD) at 450 nm within 15 min after adding the stop solution.

### 2.7 qRT‐PCR

Total RNA was extracted from rat pancreas, colon, and liver using TRIzol reagent (Invitrogen) as follows: 10 μg of total RNA was purified from organic solvents, treated with RNase‐free DNase I (Roche), and then reverse transcribed to synthesize cDNA using oligo dT primer and SuperScript II RT enzyme (Invitrogen). The cDNA was synthesized by reverse transcription using oligo dT primer and SuperScript II RT enzyme (Invitrogen). The resulting cDNA served as a template for quantitative real‐time PCR, with rp49 as the internal reference gene for RNA normalization. Reactions were carried out in triplicate on an ABI7300 equipment (Applied Biosystems) using SYBR Green, and gene expression was examined using the comparative CT approach (Applied Biosystems). AMPK: S: 5′‐GTCAAGGTGGCCGTCAAGATA‐3′, A: 5′‐CAACTTGATGATGTGCGGATG‐3′. PGC‐1α: S: 5′‐ACCTGGCGATTCTGATTATGACT‐3′, A: 5′‐CCTTTACATTGTCCACATAGCGT‐3′.

### 2.8 High‐Throughput Sequencing of 16S rDNA From Gut Microbiota

Fresh fecal samples from 6 mice per group were collected and subjected to total microbial DNA extraction using the CTAB method, with extraction quality verified by agarose gel electrophoresis and DNA concentration quantified via UV spectrophotometry. PCR amplification targeted multiple variable regions—V3‐V4 (primers 341F/805R), V4 (515F/806R), V4‐V5 (F/R primers), and archaea‐specific regions (F/R primers)—to generate amplicons, which were purified using AMPure XT beads, quantified with Qubit, and validated for library quality using the Agilent 2100 Bioanalyzer and Illumina's library kit. Libraries that met the quality standard (at least 2 nM) were combined, heated with NaOH to create single strands, and then sequenced on a NovaSeq 6000 sequencer using 250 bp paired‐end reads. After sequencing, the data were sorted by barcode, trimmed to get rid of adapter and barcode sequences, and checked for quality using the QIIME DADA2 pipeline to produce ASV (Amplicon Sequence Variant) feature sequences. These sequences were annotated to microbial species against the SILVA database, and relative abundances of taxa across samples were calculated from the resulting ASV abundance table.

### 2.9 Determination of Short‐Chain Fatty Acids by Liquid Chromatography‐Mass Spectrometry

Weigh 50 mg of sample, add sufficient 80% methanol–water, grind thoroughly, and centrifuge at 20,000 × g for 15 min at 4°C. Transfer 20 μL of the supernatant to a 1.5 mL centrifuge tube, add EDC solution and 3‐NPH for derivatization, dilute to 500 μL with the initial mobile phase (water as phase A and methanol‐acetonitrile (1:1, v/v) as phase B), vortex mix, and transfer 200 μL to an injection vial. Agilent Poroshell 120 EC‐C18 column (2.7 μm, 2.1 × 100 mm) is run chromatographically at 40°C using a 2 μL injection volume. Mass spectrometry uses multiple reaction monitoring (MRM) in negative ion mode. Compounds are identified by comparing retention times and MRM fragment ions with standards and quantified via the internal standard method; samples with concentrations exceeding the calibration curve range are reanalyzed after appropriate dilution, with results based on diluted measurements.

### 2.10 Statistical Analyses

Data analysis of gut microbiota was performed using R language software. Correlation clustering heatmap and heatmap: the data was analyzed by clustering using the pheatmap package and presented as a clustered heatmap. Correlation network graph: use the igraph package to calculate the correlation matrix between variables, and then use the correlation function to convert it into a network graph and visualize it. Alpha diversity: Use the vegan package to calculate the metrics, such as species richness, the Shannon diversity index, etc., and combine them with the plotting packages, such as ggplot2, for the visualization. Beta diversity: Use the functions in the vegan package to visualize the data in terms of PCA, PCoA, and NMDS functions for principal component, principal coordinate, and non‐metric multidimensional scaling analysis and plotting presentation. Statistical significance for weight and blood glucose levels was assessed via log‐rank testing. Independent samples *t*‐tests, multifactor ANOVA (using SPSS software), and post hoc LSD testing examined group comparisons for notable differences. Prism software was utilized in the test for data analysis as well as for graphing. α = 0.05 (or 0.01) was used as the level of significance. Statistical differences are expressed as follows: *p < 0.05; **p < 0.01; ns for not statistically significant.

## 3 Results

### 3.1 Effects of Aerobic Exercise and LBP on the Diversity of Gut Microbiota in T2DM Rats

#### 3.1.1 α‐Diversity

**FIGURE 1:**
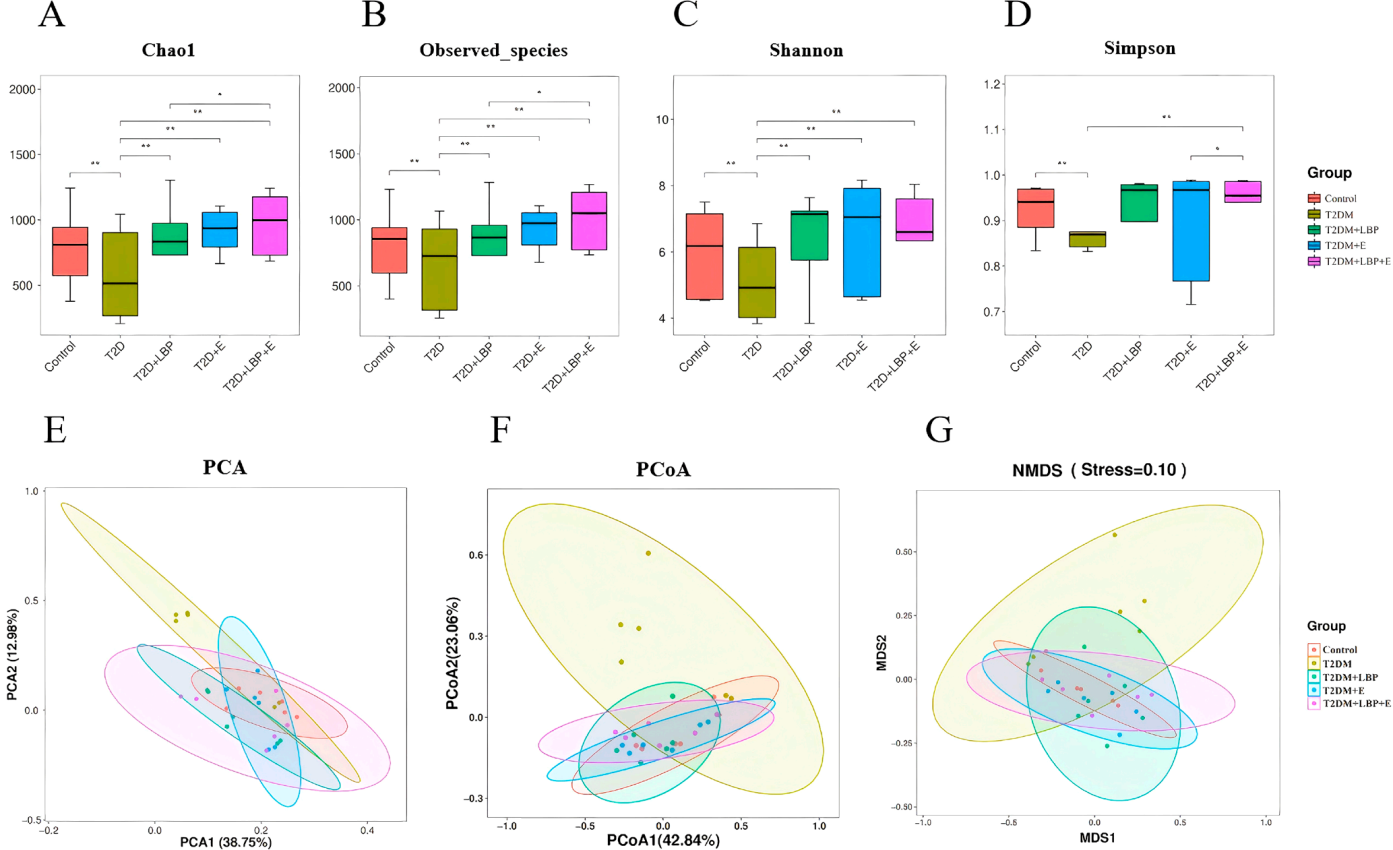
Alpha diversity analysis and Beta diversity analysis. (A, B) Chao1 and Observed_species: assessment of the number of species included in the gut microbiota. (C, D) Shannon and Simpson: assessment of gut microbiota diversity. (E) PCA: Principal Component Analysis. (F) PCoA: Principal Coordinates Analysis. (G) NMDS, nonmetric multidimensional scaling. * indicates *p* < 0.05, ** indicates *p* < 0.01.

Compared with the T2DM group, Observed species, Chao1, and Shannon were significantly higher in the Control, T2DM + LBP, T2DM + E, and T2DM + LBP + E groups (*p* < 0.01) (Figure 1A–C). Compared with the T2DM group, Simpson was significantly higher in Control and T2DM + LBP + E (*p* < 0.01) (Figure 1D).

Compared with the T2DM + LBP group, Chao1 and Observed species were significantly higher in the T2DM + LBP + E group (*p* < 0.05) (Figure 1A,B).

Compared with the T2DM + E group, Simpson was significantly higher in the T2DM + LBP + E group (*p* < 0.05) (Figure 1D).

Results of the multifactor ANOVA: The combined intervention of exercise and LBP significantly outperformed either exercise or LBP alone in influencing α‐diversity: Chao1 (*p* = 0.0013 < 0.01); Observed species (*p* = 0.0018 < 0.01); Simpson (*p* = 0.0254 < 0.05) (Figure 1A,B,D).

#### 3.1.2 β‐Diversity

According to PCA, PCoA, and NMDS, there was a significant difference in the compositional structure of the gut microbiota in the T2DM group versus the control, T2DM + LBP, T2DM + E, and T2DM + LBP + E groups (Figure 1E–G).

Based on PCA, PCoA and NMDS analyses, the compositional structure of the gut microbiota of the T2DM + LBP + E group showed significant differences from that of the T2DM + LBP and T2DM + E groups (Figure 1E–G).

### 3.2 Correlation of Gut Microbiota With SCFAs, Glucolipid Metabolism, Insulin Resistance, Inflammation, Oxidative Stress, and Molecular Pathway Indicators

#### 3.2.1 Effects of Aerobic Exercise and LBP on the Correlation of Intestinal Bacterial Phylum With SCFAs, Glucolipid Metabolism, Insulin Resistance, Inflammation, Oxidative Stress, and Molecular Pathway Indices in T2DM Rats

**FIGURE 2:**
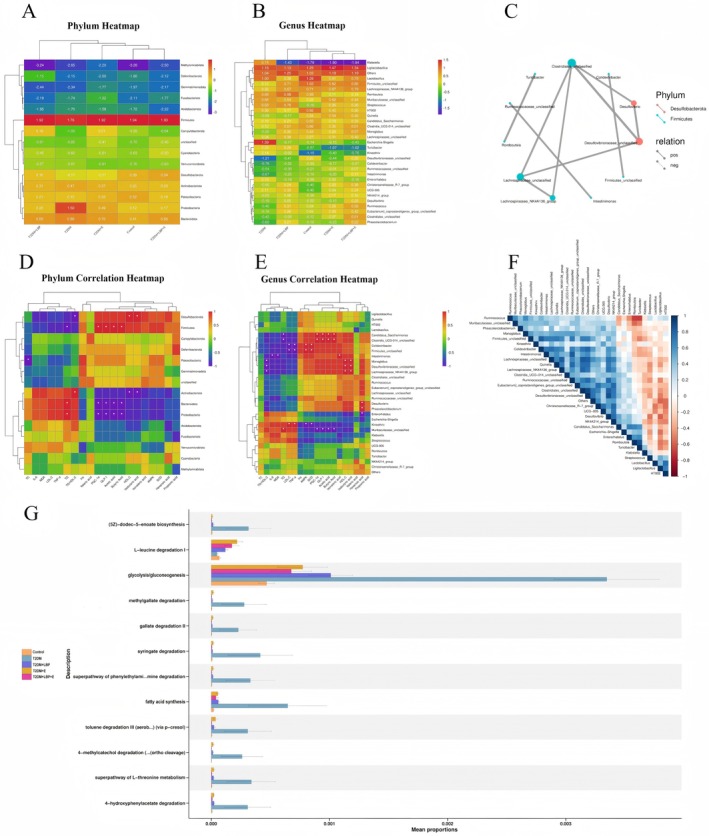
Analysis of gut microbiota abundance and its correlation with SCFAs, glucolipid metabolism, insulin resistance, inflammation, oxidative stress, and molecular pathway indicators. (A, B) Heatmap of phylum and genus. (D, E) Clustered heat maps of the correlations of phylum and genus with SCFAs, glucolipid metabolism, insulin resistance, inflammation, oxidative stress, and various indicators of molecular pathways. (C, F) Correlation network diagram of phylum and genus. (G) PICRUSt2 gut microbiota functional prediction (Pathways).

Phylum: *Firmicutes* was significantly positively correlated with butyric acid, acetic acid, GLP‐1, and PGC‐1α (*p* < 0.05), and significantly negatively correlated with TG (*p* < 0.05). Both *Proteobacteria* and *Bacteroidota* were significantly negatively correlated with acetic acid, butyric acid, GLP‐1, and PGC‐1α (*p* < 0.05), and significantly positively correlated with TG (*p* < 0.05). *Actinobacteriota* was significantly negatively correlated with isobutyric acid and HDL‐C (*p* < 0.05), and significantly positively correlated with TG/HDL‐C (*p* < 0.05). *Patescibacteria* was significantly negatively correlated with TC (*p* < 0.05). *Desulfobacterota* was significantly positively correlated with isobutyric acid and HDL‐C (*p* < 0.05), and significantly negatively correlated with TG/HDL‐C (*p* < 0.05) (Figure 2D; Tables 1 and 2).

**TABLE 1:** Correlation of gut microbiota with SCFAs.

Phylum	Genus	Acetic acid		Butyric acid		Hexanoic acid		Isobutyric acid		Isovaleric acid		Propionic acid		Valeric acid	
		a	b	a	b	a	b	a	b	a	b	a	b	a	b
*Firmicutes*		+*	0.017	+*	0.025	+	0.233	+	0.083	+	0.133	+	0.233	N	1.000
	*Ligilactobacillus*	+	0.950	+	0.942	N	1.000	+	0.783	+	0.350	−	0.517	+	0.517
	*Lactobacillus*	+	0.683	+	0.691	−	0.683	+	0.950	+	0.783	−	0.683	−	0.133
	*Firmicutes_unclassified*	+	0.083	+	0.095	+	0.450	+	0.133	+	0.083	+	0.517	+	0.950
	*Lachnospiraceae NK4A136 group*	+	0.083	+	0.078	+	0.083	+*	0.017	+	0.083	+	0.350	+	0.950
	*Romboutsia*	−	0.950	−	0.935	N	1.000	−	0.783	−	0.350	+	0.517	−	0.517
	*HT002*	+	0.783	+	0.792	−	0.45	−	0.950	+	0.783	−	0.683	−	0.783
	*Streptococcus*	−	0.233	−	0.225	−	0.783	−	0.350	−	0.133	N	1	+	0.783
	*Clostridia UCG‐014 unclassified*	+*	0.017	+*	0.022	+	0.233	+	0.083	+	0.133	+	0.233	N	1.000
	*Monoglobus*	+	0.083	+	0.090	+	0.083	+*	0.017	+	0.083	+	0.350	+	0.950
	*Quinella*	+	0.950	+	0.940	N	1.000	+	0.783	+	0.350	−	0.517	+	0.517
	*Lachnospiraceae unclassified*	+	0.517	+	0.505	+	0.083	+	0.233	+	0.350	+	0.450	+	0.350
	*Ruminococcus*	+	0.517	+	0.510	+	0.083	+	0.233	+	0.350	+	0.450	+	0.350
	*Eubacterium coprostanoligenes group unclassified*	+	0.517	+	0.525	+	0.083	+	0.233	+	0.350	+	0.450	+	0.350
	*Clostridiales_unclassified*	+	0.683	+	0.670	+	0.233	+	0.350	+	0.233	+	0.783	+	0.233
	*Christensenellaceae R‐7 group*	N	1.000	N	0.995	+	0.233	+	0.517	+	0.683	+	0.783	+	0.45
	*UCG‐005*	−	0.783	−	0.770	+	0.450	+	0.950	−	0.783	+	0.683	+	0.783
	*Phascolarctobacterium*	+	0.233	+	0.220	+*	0.017	+	0.083	+	0.233	+	0.233	+	0.683
	*NK4A214 group*	−	0.683	−	0.668	+	0.783	N	1.000	+	0.783	−	0.783	+	0.133
	*Turicibacter*	−	0.950	−	0.958	N	1.000	−	0.783	−	0.350	+	0.517	−	0.517
	*Ruminococcaceae_unclassified*	+	0.450	+	0.442	+	0.350	+	0.233	+	0.083	+	0.950	+	0.450
	*Intestinimonas*	+	0.133	+	0.125	+	0.233	+	0.083	+*	0.017	+	0.683	+	0.783
	*Kineothrix*	−	0.083	−	0.075	−	0.45	−	0.133	−	0.083	−	0.517	−	0.950
	*Colidextribacter*	+	0.083	+	0.068	+	0.450	+	0.133	+	0.083	+	0.517	+	0.950
*Proteobacteria*		−*	0.017	−*	0.019	−	0.233	−	0.083	−	0.133	−	0.233	N	1
	*Escherichia‐Shigella*	−	0.517	−	0.502	−	0.083	−	0.233	−	0.35	−	0.450	−	0.350
	*Klebsiella*	−	0.450	−	0.435	−	0.350	−	0.233	−	0.083	−	0.950	−	0.450
*Bacteroidota*		−*	0.017	−*	0.027	−	0.233	−	0.083	−	0.133	−	0.233	N	1
	*Muribaculaceae unclassified*	−*	0.017	−*	0.020	−	0.233	−	0.083	−	0.133	−	0.233	N	1
*Actinobacteriota*		−	0.083	−	0.080	−	0.083	−*	0.017	−	0.083	−	0.350	−	0.950
	*Enterorhabdus*	−	0.233	−	0.215	−*	0.017	−	0.083	−	0.233	−	0.233	−	0.683
*Patescibacteria*		+	0.350	+	0.342	+	0.950	+	0.683	+	0.517	+	0.450	+	0.517
	*Candidatus Saccharimonas*	+*	0.017	+*	0.023	+	0.233	+	0.083	+	0.133	+	0.233	N	1
*Desulfobacterota*		+	0.083	+	0.091	+	0.083	+*	0.017	+	0.083	+	0.350	+	0.950
	*Desulfovibrionaceae_unclassified*	+	0.083	+	0.942	+	0.083	+*	0.017	+	0.083	+	0.350	+	0.950
	*Desulfovibrio*	+	0.233	+	0.225	+*	0.017	+	0.083	+	0.233	+	0.233	+	0.683

*Note:* a represents correlation; b represents *p*‐value; + indicates positive correlation; − indicates negative correlation; * indicates *p* < 0.05 or 0.01 indicates significant correlation.

**TABLE 2:** Correlation of gut microbiota with glucolipid metabolism, insulin resistance, inflammation, oxidative stress, and molecular pathway indicators.

Phylum	Genus	TC		TG		HDL‐C		LDL‐C		TG/HDL‐C		Ins		GLP‐1		TNF‐α		IL‐6		AMPK		PGC‐1α		SOD		MDA	
		a	b	a	b	a	b	a	b	a	b	a	b	a	b	a	b	a	b	a	b	a	b	a	b	a	b
*Firmicutes*		−	0.35	−*	0.031	+	0.092	−	0.083	−	0.096	+	0.126	+*	0.019	−	0.088	−	0.142	+	0.078	+*	0.035	+	0.079	−	0.140
	*Ligilactobacillus*	−	0.683	−	0.965	+	0.795	−	0.450	−	0.798	+	0.342	+	0.938	−	0.455	−	0.359	+	0.444	+	0.957	+	0.446	−	0.360
	*Lactobacillus*	−	0.95	−	0.675	+	0.963	−	0.517	−	0.965	+	0.775	+	0.700	−	0.522	−	0.792	+	0.511	+	0.688	+	0.512	−	0.791
	*Firmicutes_unclassified*	−	0.233	−	0.072	+	0.145	−*	0.017	−	0.147	+	0.075	+	0.101	−*	0.022	−	0.092	+*	0.012	+	0.088	+*	0.013	−	0.091
	*Lachnospiraceae NK4A136 group*	−	0.683	−	0.099	+*	0.031	−	0.133	−	0.032	+	0.076	+	0.065	−	0.138	−	0.093	+	0.128	+	0.100	+	0.129		0.090
	*Romboutsia*	+	0.683	+	0.968	−	0.796	+	0.45	+	0.797	−	0.341	−	0.947	+	0.456	+	0.358	−	0.443	−	0.952	−	0.445	+	0.359
	*HT002*	−	0.35	−	0.775	−	0.964	−	0.450	+	0.962	+	0.774	+	0.800	−	0.452	−	0.793	+	0.444	+	0.788	+	0.447	−	0.790
	*Streptococcus*	+	0.45	+	0.241	−	0.363	+	0.083	+	0.365	−	0.125	−	0.218	+	0.087	+	0.141	−	0.077	−	0.239	−	0.079	+	0.140
	*Clostridia UCG‐014 unclassified*	−	0.35	−*	0.030	+	0.094	−	0.083	−	0.095	+	0.127	+*	0.018	−	0.089	−	0.142	+	0.076	+*	0.028	+	0.078	−	0.141
	*Monoglobus*	−	0.683	−	0.075	+*	0.030	−	0.133	−*	0.031	+	0.074	+	0.085	−	0.139	−	0.092	+	0.127	+	0.098	+	0.128	−	0.090
	*Quinella*	−	0.683	−	0.962	+	0.794	−	0.450	−	0.795	+	0.340	+	0.932	−	0.454	−	0.359	+	0.445	+	0.955	+	0.446	−	0.360
	*Lachnospiraceae unclassified*	N	1	−	0.528	+	0.246	−	0.683	−	0.247	+	0.342	+	0.499	−	0.688	−	0.358	+	0.677	+	0.532	+	0.679	−	0.357
	*Ruminococcus*	N	1	−	0.530	+	0.245	−	0.683	−	0.244	+	0.341	+	0.502	−	0.689	−	0.359	+	0.676	+	0.522	+	0.678	−	0.358
	*Eubacterium coprostanoligenes group unclassified*	N	1	−	0.508	+	0.246	−	0.683	−	0.245	+	0.343	+	0.535	−	0.687	−	0.360	+	0.678	+	0.519	+	0.677	−	0.359
	*Clostridiales_unclassified*	−	0.95	−	0.695	+	0.362	−	0.517	−	0.363	+	0.225	+	0.685	−	0.523	−	0.242	+	0.510	+	0.698	+	0.511	−	0.241
	*Christensenellaceae R‐7 group*	+	0.517	N	1.012	+	0.530	+	0.950	−	0.531	−	0.675	N	0.988	+	0.956	−	0.692	−	0.943	N	1.005	−	0.945	−	0.691
	*UCG‐005*	+	0.35	+	0.795	+	0.963	+	0.450	−	0.964	−	0.776	−	0.802	+	0.453	+	0.792	−	0.445	−	0.781	−	0.444	+	0.790
	*Phascolarctobacterium*	−	0.95	−	0.245	+	0.095	−	0.450	−	0.094	+	0.224	+	0.230	−	0.455	−	0.243	+	0.447	+	0.250	+	0.446	−	0.240
	*NK4A214 group*	+	0.95	+	0.692	N	1.014	+	0.950	N	1.015	N	0.775	−	0.702	+	0.957	−	0.793	−	0.946	−	0.680	−	0.944	−	0.791
	*Turicibacter*	+	0.683	+	0.938	−	0.796	+	0.450	+	0.797	−	0.342	−	0.969	+	0.454	+	0.358	−	0.448	−	0.945	−	0.443	+	0.359
	*Ruminococcaceae_unclassified*	−	0.683	−	0.465	+	0.246	−	0.233	−	0.245	+	0.075	+	0.438	−	0.238	−	0.091	+	0.227	+	0.457	+	0.228	−	0.090
	*Intestinimonas*	−	0.517	−	0.141	+	0.094	−	0.083	−	0.095	+	0.076	+	0.118	−	0.088	−*	0.028	+	0.079	+	0.139	+	0.077	−*	0.027
	*Kineothrix*	+	0.233	+	0.092	−	0.146	+*	0.017	+	0.145	−	0.074	−	0.085	+*	0.023	+	0.092	−*	0.010	−	0.102	−*	0.011	+	0.090
	*Colidextribacter*	−	0.233	−	0.098	+	0.147	−	0.017	−	0.146	+	0.075	+	0.079	−*	0.021	−	0.093	+*	0.013	+	0.089	+*	0.012	−	0.091
*Proteobacteria*		+	0.35	+*	0.032	−	0.095	+	0.083	+	0.094	−	0.126	−*	0.024	+	0.089	+	0.142	−	0.076	−*	0.018	−	0.078	+	0.140
	*Escherichia‐Shigella*	N	1	+	0.533	−	0.246	+	0.683	+	0.245	−	0.341	−	0.515	+	0.689	+	0.359	−	0.677	−	0.529	−	0.678	+	0.360
	*Klebsiella*	+	0.683	+	0.468	−	0.244	+	0.233	+	0.243	−	0.076	−	0.447	+	0.239	+	0.092	−	0.226	−	0.452	−	0.228	+	0.090
*Bacteroidota*		+	0.35	+*	0.035	−	0.093	+	0.083	+	0.092	−	0.125	−*	0.015	+	0.087	+	0.141	−	0.077	−*	0.029	−	0.079	+	0.140
	*Muribaculaceae unclassified*	+	0.35	+	0.033	−	0.094	+	0.083	+	0.095	−	0.127	−*	0.016	+	0.088	+	0.142	−	0.076	−*	0.025	−	0.078	+	0.141
*Actinobacteriota*		+	0.683	+	0.095	−*	0.030	+	0.133	+*	0.031	−	0.074	−	0.070	+	0.138	+	0.093	−	0.127	−	0.103	−	0.129	+	0.091
	*Enterorhabdus*	+	0.95	+	0.248	−	0.095	+	0.45	+	0.094	−	0.226	−	0.229	+	0.455	+	0.242	−	0.446	−	0.235	−	0.444	+	0.241
*Patescibacteria*		−*	0.017	−	0.365	+	0.696	−	0.233	−	0.697	+	0.509	+	0.338	−	0.238	−	0.526	+	0.228	+	0.357	+	0.227	−	0.525
	*Candidatus Saccharimonas*	−	0.35	−*	0.031	+	0.094	−	0.083	−	0.093	+	0.125	+*	0.018	−	0.089	−	0.141	+	0.078	+*	0.026	+	0.077	−	0.140
*Desulfobacterota*		−	0.683	−	0.075	+*	0.030	−	0.133	−*	0.031	+	0.075	+	0.100	−	0.139	−	0.092	+	0.129	+	0.088	+	0.128	−	0.090
	*Desulfovibrionaceae_unclassified*	−	0.683	−	0.965	+*	0.032	−	0.133	−	0.031	+*	0.076	+	0.938	−	0.138	−	0.093	+	0.128	+	0.957	+	0.127	−	0.091
	*Desulfovibrio*	−	0.95	−	0.241	+	0.095	−	0.450	−	0.094	+	0.225	+	0.218	−	0.456	−	0.243	+	0.443	+	0.239	+	0.445	−	0.240

*Note:* a represents correlation; b represents *p*‐value; + indicates positive correlation; − indicates negative correlation; * indicates *p* < 0.05 or 0.01 indicates significant correlation.

#### 3.2.2 Effects of Aerobic Exercise and LBP on the Correlation of Intestinal Bacterial Genus With SCFAs, Glucolipid Metabolism, Insulin Resistance, Inflammation, Oxidative Stress, and Molecular Pathway Indices in T2DM Rats

Genus: *Firmicutes unclassified* showed a significant negative correlation with TNF‐α and LDL‐C (*p* < 0.05), and a significant positive correlation with SOD and AMPK (*p* < 0.05). *Lachnospiraceae NK4A136 group* exhibited positive correlations with isobutyric acid and HDL‐C (*p* < 0.05), and a negative correlation with TG/HDL‐C (*p* < 0.05). *Clostridia UCG‐014 unclassified* was positively correlated with butyric acid, acetic acid, and PGC‐1α (*p* < 0.05), and negatively correlated with TG and GLP‐1 (*p* < 0.05). *Monoglobus* demonstrated positive associations with isobutyric acid and HDL‐C (*p* < 0.05), and a negative association with TG/HDL‐C (*p* < 0.05). *Phascolarctobacterium* showed a positive correlation with hexanoic acid (*p* < 0.05). *Intestinimonas* was positively correlated with isovaleric acid (*p* < 0.05) and negatively correlated with IL‐6 and MDA (*p* < 0.05). *Kineothrix* exhibited positive correlations with LDL‐C and TNF‐α (*p* < 0.05), and negative correlations with SOD and AMPK (*p* < 0.05). *Colidextribacter* showed negative correlations with LDL‐C and TNF‐α (*p* < 0.05), and positive correlations with SOD and AMPK (*p* < 0.05). *Muribaculaceae unclassified* was negatively correlated with acetic acid, butyric acid, and PGC‐1α (*p* < 0.05), and positively correlated with GLP‐1 and TG (*p* < 0.05). *Enterorhabdus* demonstrated a negative correlation with hexanoic acid (*p* < 0.05). *Candidatus Saccharimonas* showed positive correlations with acetic acid, butyric acid, and PGC‐1α (*p* < 0.05), and negative correlations with GLP‐1 and TG (*p* < 0.05). *Desulfovibrionaceae unclassified* was positively correlated with isobutyric acid, HDL‐C, and Ins (*p* < 0.05). *Desulfovibrio* exhibited a positive correlation with hexanoic acid (*p* < 0.05) (Figure 2E; Tables 1 and 2).

#### 3.2.3 PICRUSt2 Gut Microbiota Functional Prediction

Glucose Metabolism Pathway: As the core pathway of glucose metabolism, the average proportion of glycolysis/gluconeogenesis in the T2DM group was significantly higher than that in the Control group, suggesting aggravated glucose metabolism disorders under the diabetic state. Compared with the T2DM group, the abundance of this pathway in the T2DM + LBP group, T2DM + E group, and T2DM + LBP + E group decreased in a gradient manner, with the most significant decrease observed in the T2DM + LBP + E group. This indicates that *Lycium barbarum* polysaccharides (LBP) combined with exercise can effectively regulate the glucose metabolism pathway and improve glucose metabolism disorders (Figure 2G).

Lipid Metabolism Pathway: The abundance of the fatty acid synthesis pathway in the T2DM group showed an increasing trend compared with the Control group, reflecting lipid metabolism abnormalities under the diabetic state. The T2DM + LBP group, T2DM + E group, and T2DM + LBP + E group all exerted different degrees of inhibitory effects on the abundance of this pathway, and the T2DM + LBP + E group exhibited the most prominent regulatory effect. This suggests that the intervention (LBP combined with exercise) can improve lipid metabolism disorders by regulating the lipid metabolism pathway (Figure 2G).

Other Metabolic Pathways: The average proportions of other metabolic pathways, including (5Z)‐dodec‐5‐enoate biosynthesis, L‐leucine degradation I, methylgallate degradation, gallate degradation II, syringate degradation, the superpathway of phenylethylamine degradation, toluene degradation III (aerobic; via p‐cresol), 4‐methylcatechol degradation (…ortho cleavage), the superpathway of L‐threonine metabolism, and 4‐hydroxyphenylacetate degradation, were generally low. However, the abundance of these pathways in the T2DM group was still relatively prominent compared with other groups. It can be seen that each intervention group (T2DM + LBP, T2DM + E, T2DM + LBP + E) also exerted a certain regulatory effect on these pathways, which reflects the effect of the interventions (Figure 2G).

### 3.3 Effects of Aerobic Exercise and LBP on the Abundance of Gut Microbiota in T2DM Rats

**FIGURE 3:**
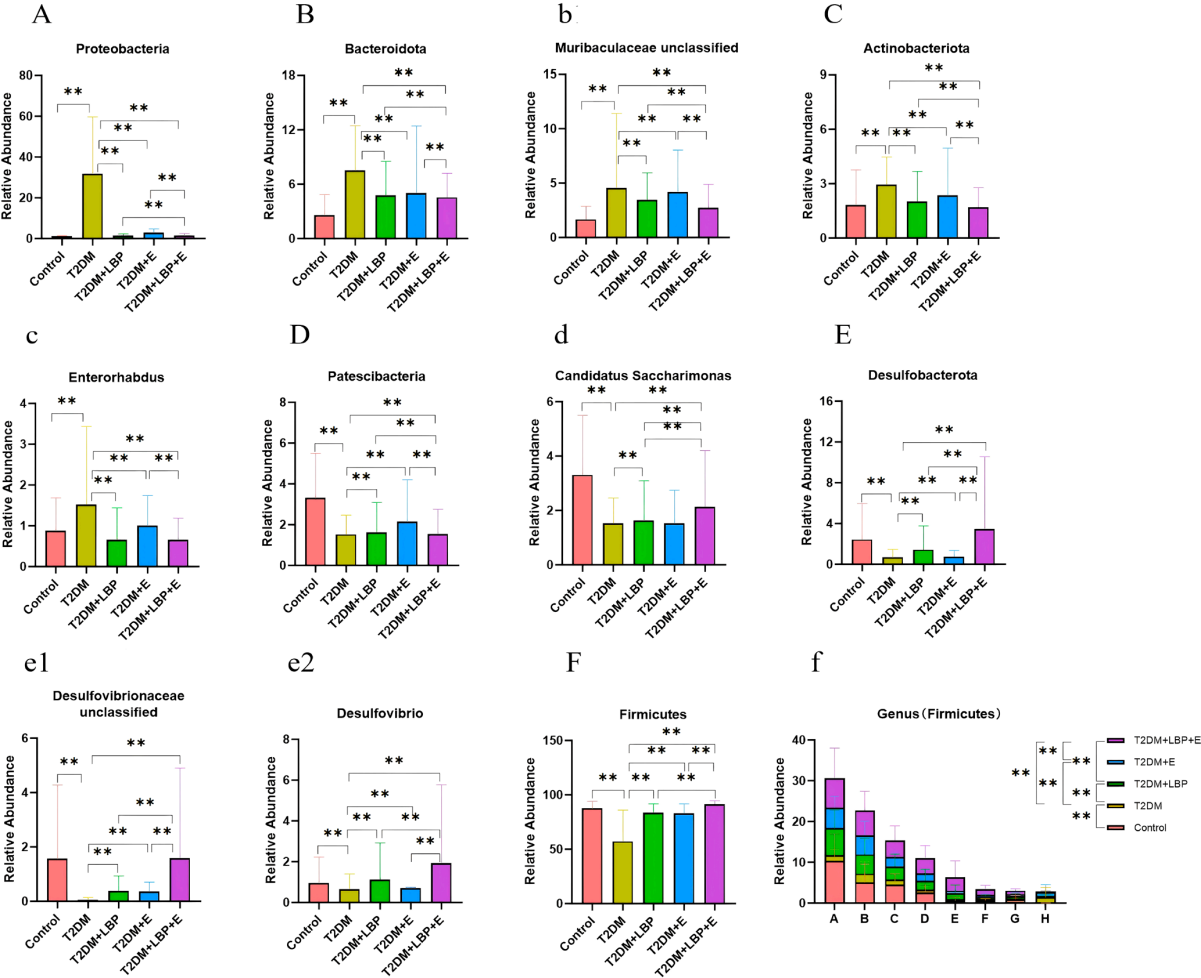
Effects of aerobic exercise and LBP on the abundance of gut microbiota in T2DM rats. (A–F) Denotes the comparison of differences in the abundance of gut microbiota phylum in each group. (b) Affiliation to Bacteroidota indicates a comparison of differences in genus abundance of enteric flora across groups. (c) Affiliation to Actinobacteriota indicates a comparison of differences in genus abundance of enteric flora across groups. (d) Affiliation to Patescibacteria indicates a comparison of differences in genus abundance of enteric flora across groups. (e1, e2) Affiliation to Desulfobacterota indicates a comparison of differences in genus abundance of enteric flora across groups. (f) Affiliation to the Firmicutes indicates differences in the abundance of gut microbiota genera between groups. A, *Firmicutes_unclassified*; B, *Lachnospiraceae_NK4A136_group*; C, *Clostridia_UCG‐014_unclassified*; D, *Monoglobus*; E, *Phascolarctobacterium*; F, *Intestinimonas*; G, *Colidextribacter*; H, *Kineothrix*. * indicates *p* < 0.05, ** indicates *p* < 0.01.

Phylum: Compared with the T2DM group, *Firmicutes, Desulfobacterota*, and *Patescibacteria* were significantly higher in the Control, T2DM + LBP, T2DM + E, and T2DM + LBP + E groups (*p* < 0.01), and *Proteobacteria*, *Bacteroidota*, and *Actinobacteriota* were significantly lower (*p* < 0.01) (Figures 2A and 3A–F). Genus: Compared to the T2DM group, Control, T2DM + LBP, T2DM + E, and T2DM + LBP + E groups were Lachnospiraceae NK4A136 group, Firmicutes unclassified, *Clostridia UCG‐014 unclassified*, *Monoglobus*, *Phascolarctobacterium*, *Intestinimonas*, *Colidextribacter*, *Candidat*us Saccharimonas, *Desulfovibrionaceae unclassified*, and *Desulfovibrio* were significantly higher (*p* < 0.01), and *Kineothrix*, *Muribaculaceae unclassified*, and *Enterorhabdus* were significantly lower (*p* < 0.01) (Figures 2B and 3b–d,e1,e2,f).

Phylum: Compared with the T2DM + LBP group, *Firmicutes*, *Patescibacteria*, and *Desulfobacterota* were significantly higher in the T2DM + LBP + E group (*p* < 0.01), and *Proteobacteria*, *Bacteroidota*, and *Actinobacteriota* were significantly lower (*p* < 0.01) (Figures 2A and 3A–F). Genus: Compared with the T2DM + LBP group, the T2DM + LBP + E group had Lachnospiraceae NK4A136 group, *Firmicutes unclassified*, *Clostridia UCG‐014 unclassified*, *Monoglobus*, *Phascolarctobacterium*, *Intestinimonas*, *Colidextribacter*, *Candidatus Saccharimonas*, *Desulfovibrionaceae unclassified*, and *Desulfovibrio* significantly higher (*p* < 0.01) and significantly lower (*p* < 0.01) for *Kineothrix* and *Muribaculaceae unclassified* (Figures 2B and 3b–e1,e2,f).

Phylum: Compared with the T2DM + E group, the levels of *Firmicutes*, *Patescibacteria*, and *Desulfobacterota* in the T2DM + LBP + E group were significantly increased (*p* < 0.01). *Proteobacteria*, *Bacteroidota*, and *Actinobacteriota* were significantly decreased (*p* < 0.01) (Figures 2A and 3A–F). Genus: Compared with the T2DM + E group, Lachnospiraceae NK4A136 group, *Firmicutes unclassified*, *Clostridia UCG‐014 unclassified*, *Monoglobus* in the T2DM + LBP + E group, *Phascolarctobacterium*, *Intestinimonas*, *Colidextribacter*, *Desulfovibrionaceae unclassified*, and *Desulfovibrio* were significantly elevated (*p* < 0.01), and *Kineothrix*, *Muribaculaceae unclassified*, and *Enterorhabdus* were significantly lower (*p* < 0.01) (Figures 2B and 3b,c,e1,e2,f).

Results of the multifactorial ANOVA: The combined intervention of exercise and LBP significantly outperformed either exercise or LBP alone in affecting gut microbiota abundance: *Proteobacteria* (*p* = 0.0073 < 0.01); *Bacteroidota* (*p* = 0.0092 < 0.01); *Muribaculaceae unclassified* (*p* = 0.0035 < 0.05); *Actinobacteriota* (*p* = 0.0009 < 0.01); *Enterorhabdus* (*p* = 0.0061 < 0.01); *Patescibacteria* (*p* = 0.0047 < 0.05); *Candidat*us Saccharimonas (*p* = 0.0085 < 0.01); *Desulfobacterota* (*p* = 0.0022 < 0.01); *Desulfovibrionaceae unclassified* (*p* = 0.0058 < 0.05); *Desulfovibrio* (*p* = 0.0011 < 0.01); *Firmicutes* (*p* = 0.0079 < 0.01); *Firmicutes_unclassified* (*p* = 0.0039 < 0.05); *Lachnospiraceae_NK4A136_group* (*p* = 0.0006 < 0.01); *Clostridia_UCG‐014_unclassified* (*p* = 0.0068 < 0.01); *Monoglobus* (*p* = 0.0029 < 0.05); Phascolarctobacterium (*p* = 0.0081 < 0.01); *Intestinimonas* (*p* = 0.0043 < 0.01); *Colidextribacter* (*p* = 0.0015 < 0.05); *Kineothrix* (*p* = 0.0052 < 0.01) (Figure 3A,B,b,C,c,D,E,e1,e2,F,f1,f2).

### 3.4 Effects of Aerobic Exercise and LBP on SCFAs in T2DM Rats

**FIGURE 4:**
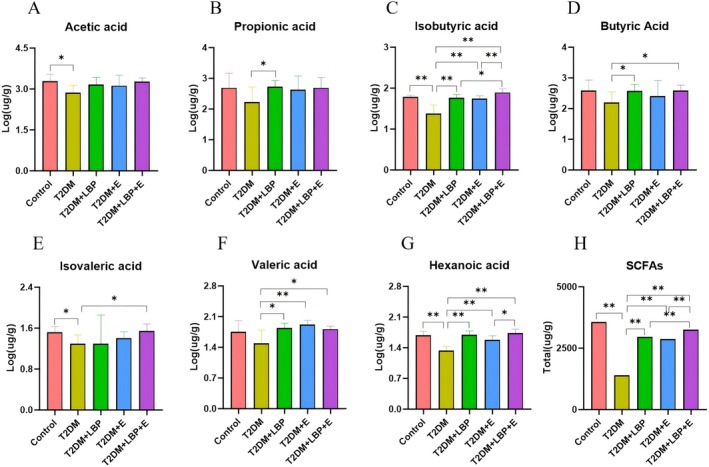
Effects of aerobic exercise and LBP on SCFAs in T2DM rats. (A) Indicates the comparison of the difference of Acetic acid in each group. (B) Indicates the comparison of the difference of Propionic acid in each group. (C) Indicates the comparison of the differences of Isobutyric acid in each group. (D) Indicates the difference of Butyric acid in each group. (E) Indicates the difference of Isovaleric acid in each group. (F) Indicates the difference of Valeric acid in each group. (G) Indicates the difference of Hexanoic acid in each group. (H) Comparison of total SCFAs by group. * indicates *p* < 0.05, ** indicates *p* < 0.01.

Compared with the T2DM group, isobutyric acid, acetic acid, hexanoic acid, isovaleric acid, and total SCFAs were significantly higher in the control group (*p* < 0.05 or *p* < 0.01) (Figure 4A,C,E,G,H). Propionic acid, isobutyric acid, butyric acid, valeric acid, hexanoic acid, and total SCFAs were significantly increased in the T2DM + LBP group (*p* < 0.01) (Figure 4B–D,F–H). Isobutyric acid, hexanoic acid, valeric acid, and total SCFAs were significantly increased in the T2DM + E group (*p* < 0.01) (Figure 4C,F–H). Isobutyric acid, isovaleric acid, butyric acid, hexanoic acid, valeric acid, and total SCFAs were significantly increased in the T2DM + LBP + E group (*p* < 0.05 or *p* < 0.01) (Figure 4C–H).

Compared with the T2DM + LBP group, isobutyric acid and total SCFAs were significantly higher in the T2DM + LBP + E group (*p* < 0.05 or *p* < 0.01) (Figure 4C,H).

Compared with the T2DM + E group, isobutyric acid, hexanoic acid, and total SCFAs were significantly higher in the T2DM + LBP + E group (*p* < 0.05 or *p* < 0.01) (Figure 4C,G,H).

Results of the multifactor ANOVA: The combined intervention of exercise and LBP significantly outperformed either exercise or LBP alone in affecting short‐chain fatty acids: isobutyric acid (*p* = 0.0041 < 0.05); butyric acid (*p* = 0.0413 < 0.05); isovaleric acid (*p* = 0.0497 < 0.05); hexanoic acid (*p* = 0.0271 < 0.05); total SCFAs (*p* = 0.0095 < 0.01) (Figure 4C–E,G,H).

### 3.5 Effects of Aerobic Exercise and LBP on Glycolipid Metabolism, Insulin Resistance and Inflammatory Response in T2DM Rats

**FIGURE 5:**
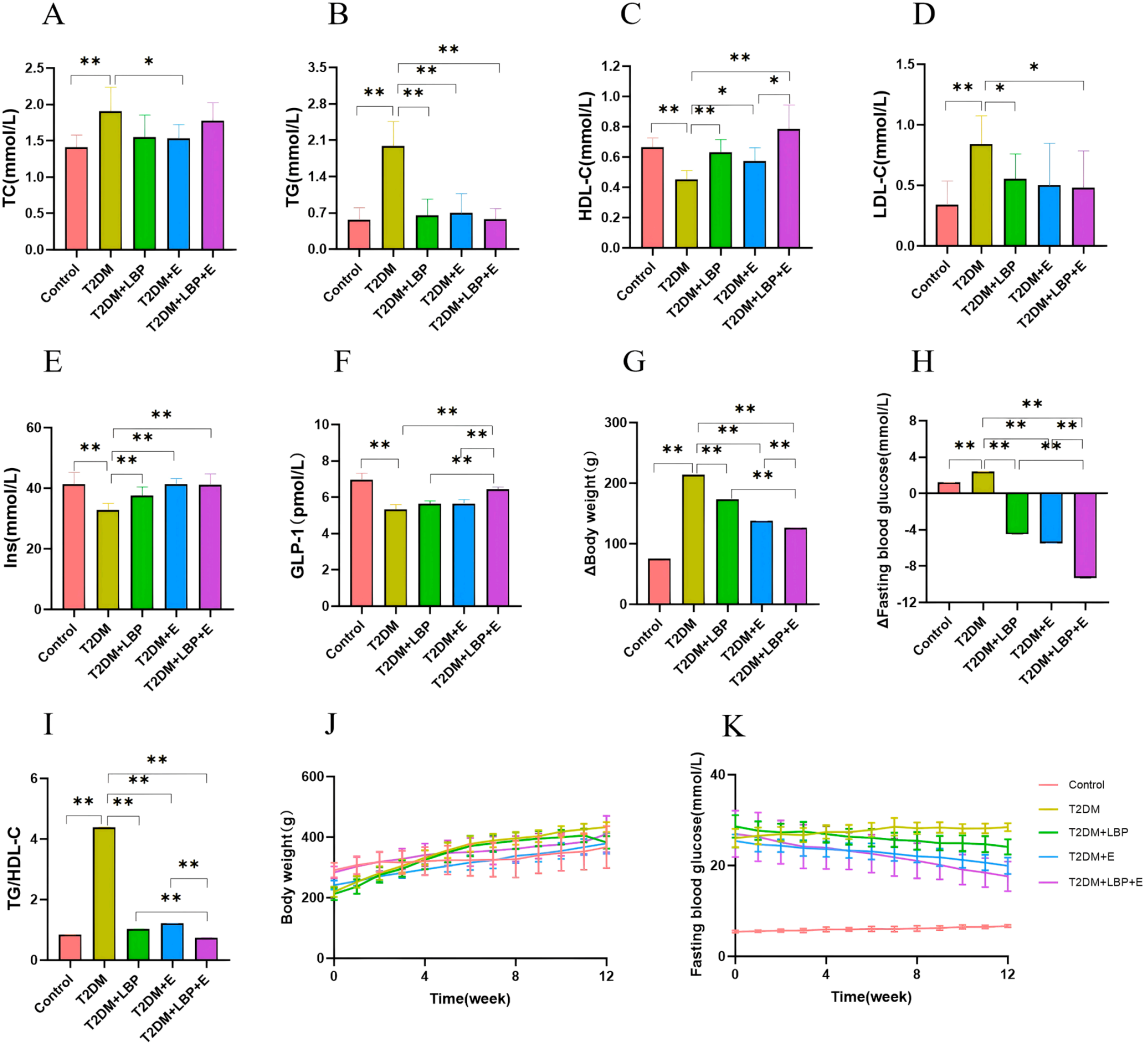
Effects of aerobic exercise and LBP on glycolipid metabolism and insulin resistance in T2DM rats. (A) Indicates the difference of TC (total cholesterol) in each group. (B) Comparison of differences in TG (triglyceride) between groups. (C, D) Comparison of HDL‐C/LDL‐C (high/low density lipoprotein) in each group. (E) Comparison of the differences in INS (insulin) between groups. (F) Comparison of GLP‐1 (glucagon peptide) by group. (G) Indicates the difference of weight change in each group. (H) Indicates the difference of fasting blood glucose change in each group. (I) Indicates the difference of TG/HDL‐C in each group, which reflects the level of insulin resistance. (J) Indicates the trend of weight change in each group. (K) Indicates the trend of fasting blood glucose changes in each group. * indicates *p* < 0.05, ** indicates *p* < 0.01.

**FIGURE 6:**
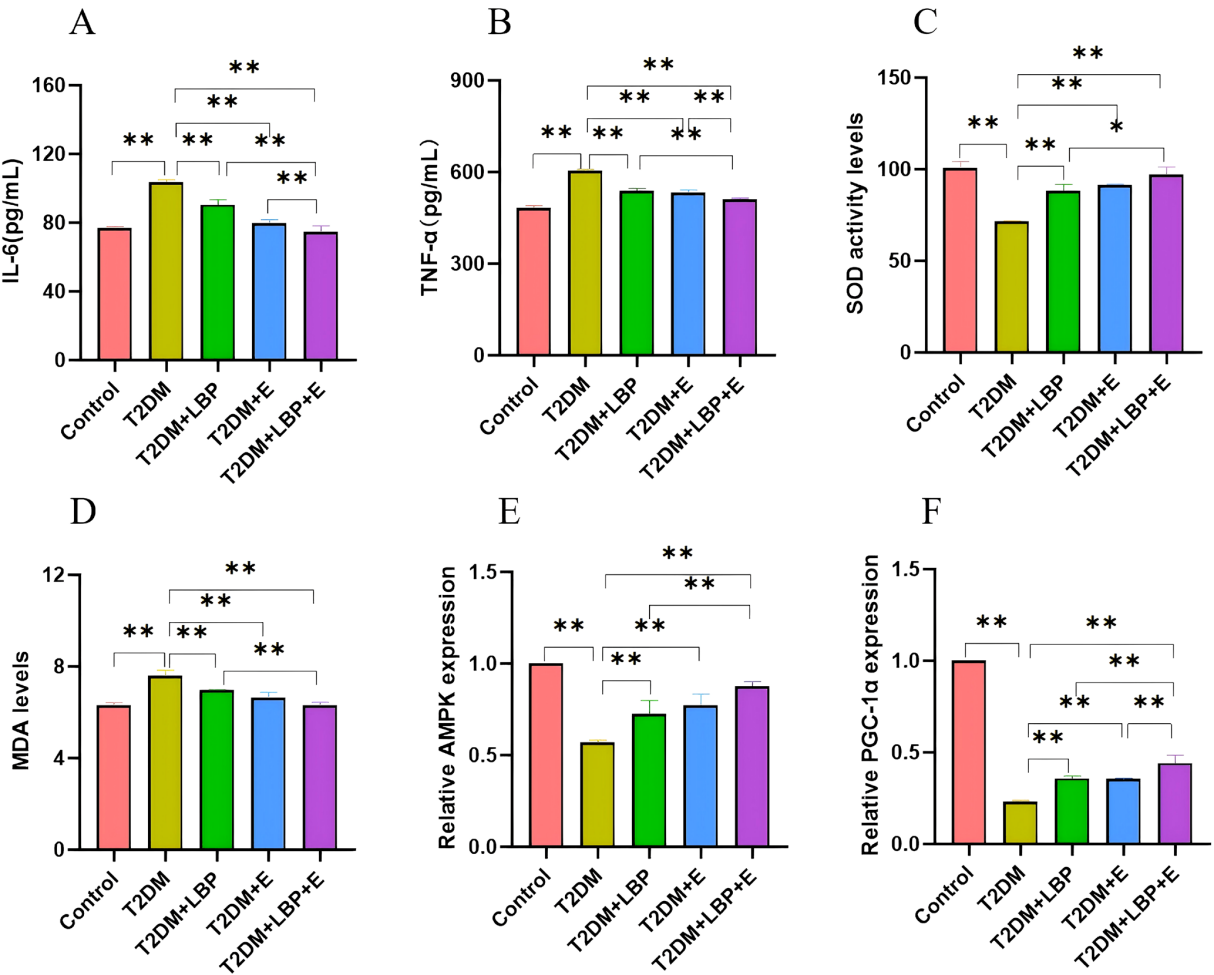
Effects of aerobic exercise and LBP on inflammatory responses, oxidative stress, and molecular pathways in T2DM rats. (A) Indicates the comparison of the difference of IL‐6 (Interleukin‐6) in each group. (B) Indicates the comparison of differences in TNF‐α (Tumor Necrosis Factor‐α) in each group. (C) Differences in SOD (Superoxide Dismutase) between groups. (D) Comparison of MDA (Malondialdehyde) in each group. (E) Comparison of AMPK (AMP‐activated protein kinase) in each group. (F) Comparison of PGC‐1α (Peroxisome proliferator‐activated receptor‐gamma co‐activator 1‐alpha) in each group. * indicates *p* < 0.05, ** indicates *p* < 0.01.

Compared with the T2DM group, LDL‐C, TC, TG, fasting blood glucose change, weight change, TG/HDL‐C, TNF‐α, and IL‐6 were significantly lower (*p* < 0.01), and HDL‐C, Ins, and GLP‐1 were significantly higher (*p* < 0.01) in the control group (Figures 5A–I and 6A,B). The T2DM + LBP group had significantly lower TG, LDL‐C, TG/HDL‐C, IL‐6, TNF‐α, and weight change (*p* < 0.05 or *p* < 0.01), and significantly higher HDL‐C, Ins, and fasting blood glucose change (*p* < 0.01) (Figures 5B–E,G–I and 6A,B). The T2DM + E group had significantly lower TC, TG, TG/HDL‐C, IL‐6, TNF‐α, and weight change (*p* < 0.05 or *p* < 0.01) and significantly higher HDL‐C, Ins, and fasting blood glucose change (*p* < 0.05 or *p* < 0.01) (Figures 5A,B,D,E,G–I and 6A,B). The T2DM + LBP + E group had significantly lower TG, LDL‐C, weight change, TG/HDL‐C, IL‐6, and TNF‐α (*p* < 0.05 or *p* < 0.01), and significantly higher HDL‐C, Ins, GLP‐1, and fasting blood glucose change (*p* < 0.01) (Figures 5B–I and 6A,B).

Compared to the T2DM + LBP group, weight change, TG/HDL‐C, TNF‐α, and IL‐6 were significantly lower (*p* < 0.01), and GLP‐1 and fasting blood glucose change were significantly higher (*p* < 0.01) in the T2DM + LBP + E group (Figures 5F,H,I and 6A,B).

Compared with the T2DM + E group, weight change, TG/HDL‐C, TNF‐α, and IL‐6 were significantly lower (*p* < 0.01), and HDL‐C, GLP‐1, and fasting blood glucose change were significantly higher (*p* < 0.05 or *p* < 0.01) in the T2DM + LBP + E group (Figures 5C,F,H,I and 6A,B).

Long‐term diabetes mellitus elevated weight and blood glucose levels in rats, which were ameliorated by aerobic exercise and LBP intervention (Figure 5J,K).

Results of the multifactorial ANOVA: The combined intervention of exercise and LBP significantly outperformed either exercise or LBP alone in improving glucose and lipid metabolism, insulin resistance, and inflammatory responses: TG (*p* = 0.0496 < 0.05); HDL‐C (*p* = 0.0089 < 0.01); LDL‐C (*p* = 0.0371 < 0.05); GLP‐1 (*p* = 0.0028 < 0.05); TG/HDL‐C (*p* = 0.0019 < 0.05); IL‐6 (*p* = 0.0006 < 0.01); TNF‐α (*p* = 0.0017 < 0.05) (Figures 5B–D,F,G,I and 6A,B).

### 3.6 Effects of Aerobic Exercise and LBP on Oxidative Stress and Molecular Pathways in T2DM Rats

Compared with the T2DM group, the control group showed a significant decrease in the levels of MDA (*p* < 0.01) and a significant increase in the levels of SOD vigor, the relative expression of AMPK, and the relative expression of PGC‐1α (*p* < 0.01) (Figure 6C–F). The levels of MDA were significantly decreased (*p* < 0.01), and the levels of SOD vigor, relative expression of AMPK, and relative expression of PGC‐1α were significantly increased (*p* < 0.01) in the T2DM + LBP group (Figure 6C–F). The levels of MDA were significantly decreased (*p* < 0.01), and the levels of SOD vigor, relative expression of AMPK, and relative expression of PGC‐1α were significantly increased (*p* < 0.01) in the T2DM + E group (Figure 6C–F). The levels of MDA were significantly decreased in the T2DM + LBP + E group (*p* < 0.01), and the levels of SOD vigor, relative expression of AMPK, and relative expression of PGC‐1α were significantly increased (*p* < 0.01) (Figure 6C–F).

Compared with the T2DM + LBP group, the T2DM + LBP + E group showed a significant decrease in the levels of MDA (*p* < 0.01) and a significant increase in the levels of SOD vitality, the relative expression of PGC‐1α, and the relative expression of AMPK (*p* < 0.05 or *p* < 0.01) (Figure 6C–F).

Compared with the T2DM + E group, the T2DM + LBP + E group showed significantly higher relative expression of PGC‐1α (*p* < 0.01) (Figure 6F).

Results of the multifactor ANOVA: The combined intervention of exercise and LBP significantly outperformed either exercise or LBP alone in their effects on molecular pathways and oxidative stress: SOD (*p* = 0.0332 < 0.05); MDA (*p* = 0.0269 < 0.05); AMPK (*p* = 0.0473 < 0.05); PGC‐1α (*p* = 0.0078 < 0.01) (Figure 6C–F).

## 4 Discussion

### 4.1 Effects of T2DM on Gut Microbiota

Gut microbiota play a crucial role in human health, and their ecological dysregulation is associated with various pathologic processes (Han et al. 2021). Patients with T2DM often exhibit intestinal dysbiosis and dysfunction involving multiple organs (Liu et al. 2022), and maintaining or reestablishing microbial diversity is key to preventing gut homeostasis imbalances linked to such dysbiosis. Gut microbiota may contribute to T2DM via insulin resistance (IR) and disrupted lipid metabolism (Pedersen et al. 2016). Notably, obesity—characterized by abnormal visceral fat accumulation—is a major risk factor for T2DM (Frank et al. 2021), as fat accumulation exacerbates IR, suppresses insulin secretion, reduces cellular glucose uptake, elevates blood glucose, and ultimately induces T2DM (Henao‐Mejia et al. 2012; Zheng et al. 2018).

Short‐chain fatty acids (SCFAs), metabolites produced by intestinal microbes via indigestible polysaccharide degradation, exhibit a reciprocal regulatory relationship with the gut microbiota: the microbiota shapes SCFA composition and metabolism, while SCFAs in turn modulate microbial diversity, homeostasis, and proliferation (Yi et al. 2025). Disturbed SCFAs metabolism markedly drives hepatocellular lipogenesis and abnormal triglyceride deposition in adipose tissue (Palmas et al. 2021). Specifically, SCFAs regulate lipid metabolism through two pathways: they inhibit the activity of key fatty acid synthesis enzymes and activate thermogenesis‐related signaling pathways, ultimately suppressing lipid synthesis, promoting energy consumption, and reducing fat deposition (Li and Guo 2023). In addition, one of the targets of SCFAs‐induced signaling is glucose homeostasis (Mokkala et al. 2020). One acknowledged modulator of glucose homeostasis is GLP‐1. Notably, administration of SCFAs via the rectal route significantly enhanced GLP‐1 secretion levels (Aragón‐Vela et al. 2021), a finding that provides direct evidence for a physiological regulatory relationship between the two (Peng et al. 2022).

Inflammation and elevated pro‐inflammatory cytokines (e.g., IL‐6, TNF‐α) are hallmarks of T2DM. Alterations in the gut microbial ecosystem can induce a pro‐inflammatory state in adipose tissue (Yehualashet and Yikna 2021), while SCFAs inhibit inflammation and modulate metabolic/immune homeostasis (He et al. 2020). Dysregulated gut microbiota reduce SCFAs biosynthesis, weakening the anti‐inflammatory defense of intestinal tissues—a process considered critical for inducing intestinal inflammation (Ma et al. 2019). SCFAs also promote repair of damaged mucosal tissues by downregulating pro‐inflammatory cytokine expression, further suppressing inflammatory responses (Koh et al. 2016; Sawicki et al. 2017).

Probiotics inhibit harmful bacterial growth, enhance intestinal barrier function, improve natural killer cell cytotoxicity, and reduce inflammatory cytokine release (La Fata et al. 2018). While pathogenic bacteria account for a small fraction of gut microbiota (Bäumler and Sperandio 2016), inflammation can induce their virulence gene expression and promote mucosal colonization (Perdijk et al. 2024). In the present study, T2DM rats had significantly lower overall gut microbiota abundance than normal rats, with increased abundances of *Kineothrix* (*Firmicutes*), *Escherichia‐Shigella* and *Klebsiella* (*Proteobacteria*), *Muribaculaceae unclassified* (*Bacteroidota*), and *Enterorhabdus* (*Actinobacteriota*)—suggesting these may act as pathogenic taxa in T2DM. T2DM also induced systemic inflammation, enhanced host cell lipogenesis and triglyceride accumulation, and disrupted glucose homeostasis, consistent with previous findings. Additionally, SCFAs levels depend heavily on commensal microbiota ratios (Ratajczak et al. 2019), and commensal dysbiosis alters SCFA production balance. Thus, in‐depth analysis of T2DM gut microbiota structure and its correlation with glucose/lipid metabolism indicators will provide a critical scientific basis for elucidating the microbiota's role in T2DM pathophysiology.

The most abundant intestinal phyla were *Bacteroidota*, *Firmicutes*, and *Actinobacteriota*: *Bacteroidota* primarily produces propionate and acetate, while Firmicutes uses butyrate as its main metabolic end product (den Besten et al. 2013). Other SCFAs include hexanoic, valeric, isovaleric, and isobutyric acids. In this study, T2DM rats showed no significant changes in butyrate, propionate, or valerate levels, but markedly downregulated hexanoic, isovaleric, isobutyric, and acetic acid levels—suggesting microbiota mediating the metabolism of these four SCFAs may drive T2DM‐related metabolic disorders. Specifically: *Unclassified Clostridia UCG‐014* (*Firmicutes*), *Muribaculaceae unclassified* (*Bacteroidota*), and *Candidatus Saccharimonas* (*Patescibacteria*) were significantly associated with acetic acid; *Lachnospiraceae NK4A136 group*, *Monoglobus* (*Firmicutes*), and *Desulfovibrionaceae unclassified* (*Desulfobacterota*) with isobutyric acid; *Intestinimonas* (*Firmicutes*) with isovaleric acid; *Phascolarctobacterium* (*Firmicutes*), *Enterorhabdus* (*Actinobacteriota*), and *Desulfovibrio* (*Desulfobacterota*) with hexanoic acid.

This provides direct evidence for our above speculations. Additionally, problems with glycolipid metabolism caused by T2DM have been linked to the previously mentioned imbalance between microbiota and SCFAs, while inflammation control has been connected to *Firmicutes unclassified* and *Colidextribacter*. Moreover, nutritional modification of the human microbiota has demonstrated numerous health advantages for the host. To prevent or mitigate the onset of diabetes in at‐risk groups, straightforward treatment methods focused on reducing chronic inflammation and insulin resistance are urgently required (Sowmiya and Silambanan 2023).

### 4.2 Effects of LBP on T2DM

Polysaccharides exhibit diverse biological activities (e.g., immunoregulation, metabolic regulation) that are closely linked to gut microbiota (Guo et al. 2023). For instance, dendrobium polysaccharides promote niacin production by Bacteroidota to activate intestinal GPR109a, enhancing intestinal barrier function and thereby improving IR in T2DM (Pi et al. 2024). Similarly, Ganoderma lucidum polysaccharides modulate gut microbiota structure in diabetic mice, significantly reducing the *Firmicutes*/*Bacteroidota* (F/B) ratio and regulating lipid metabolism (Ren et al. 2021). Chronic inflammation is tightly associated with IR and T2DM, and anti‐inflammatory polysaccharides alleviate inflammation by inhibiting pro‐inflammatory cytokine production and modulating intestinal‐systemic immune responses (Pi et al. 2024). LBP possesses strong anti‐inflammatory properties and enhances immune function (Cao, Wang, Gong, et al. 2022). For example, its arabinogalactoside isoform mitigates chronic inflammation via metabolome modulation (Cao, Wang, Ai, et al. 2022). Polysaccharides are ultimately metabolized into SCFAs, which alter gut microbiota ecology by reshaping the ratio of specific microorganisms (Wang et al. 2018). Additionally, gut microbial enzymes decompose microbe‐associated carbohydrates into simpler, host‐ and microbe‐assimilable molecules, increasing microbial diversity and abundance (Yi et al. 2025). Thus, bioactive components like LBP hold significant potential for regulating intestinal microecological balance, and their disease‐related mechanisms warrant in‐depth investigation (Zhou et al. 2022).

Polysaccharides exert hypolipidemic effects by lowering blood lipids (e.g., cholesterol, triglycerides) (Pi et al. 2024). In the present study, LBP increased HDL‐C and decreased TC, TG, and LDL‐C in T2DM rats. It also reduced IL‐6, TNF‐α, and blood glucose levels while increasing INS and GLP‐1 levels—consistent with findings that Ganoderma lucidum polysaccharides alleviate inflammation and lower blood glucose in diabetic mice (Ma et al. 2022). Polysaccharides can enable the gut microbiota to be in equilibrium (Pi et al. 2024). LBP further restored gut microbiota equilibrium: T2DM rats treated with LBP exhibited significantly higher gut microbial biodiversity than untreated T2DM rats. While an elevated F/B ratio is typically linked to obesity and gut dysbiosis (Zhang et al. 2020), the T2DM group in this study had a significantly lower F/B ratio than the LBP group—suggesting severe inflammation in T2DM rats, where *Bacteroidota* may play a pathogenic role. LBP ameliorated this response by regulating gut microbiota composition: it significantly increased the relative abundances of *Lachnospiraceae NK4A136 group*, *Firmicutes unclassified*, *Clostridia UCG‐014 unclassified*, *Monoglobus*, *Phascolarctobacterium*, *Intestinimonas*, *Colidextribacter*, *Candidatus Saccharimonas*, and *Desulfovibrionaceae unclassified*, while reducing those of *Kineothrix*, *Muribaculaceae unclassified*, and *Enterorhabdus*. Collectively, LBP enriched beneficial bacteria and depleted harmful bacteria, protecting the host from damage by pathogenic microbes and their metabolites.

Gut microbiota dysbiosis contributes to numerous metabolic diseases, making microbiota remodeling critical for host health (Tanca et al. 2017). We therefore explored correlations between gut microbiota and biochemical indices: TG was negatively correlated with *Clostridia UCG‐014 unclassified* and *Candidatus Saccharimonas*, but positively correlated with *Muribaculaceae unclassified*; HDL‐C was positively correlated with *Lachnospiraceae NK4A136 group*, *Monoglobus*, and *Desulfovibrionaceae unclassified*; LDL‐C was negatively correlated with *Colidextribacter* and *Firmicutes unclassified*, but positively correlated with *Kineothrix* (suggesting these taxa are linked to lipid metabolism); TG/HDL‐C was negatively correlated with *Lachnospiraceae NK4A136 group* and *Monoglobus*; GLP‐1 was positively correlated with *Clostridia UCG‐014 unclassified* and *Candidatus Saccharimonas*, but negatively correlated with *Muribaculaceae unclassified* (indicating involvement in glucose control and IR); IL‐6 was negatively correlated with *Monoglobus* and *Intestinimonas*; TNF‐α was negatively correlated with *Colidextribacter* and *Firmicutes unclassified*, but positively correlated with *Kineothrix* (supporting roles in inflammatory control).

Furthermore, this study discovered that LBP can reduce inflammation and control gut microbiota proportions while also increasing SCFAs production. Propionate, acetate, and butyrate prevent diet‐induced obesity and insulin resistance, while propionate and butyrate induce gut hormones and reduce food intake (Lin et al. 2012). Butyrate and propionate activate intestinal gluconeogenesis through complementary mechanisms (de Vadder et al. 2014). In our study, we found that LBP significantly increased the amount of butyric acid. And in combination with the above results, involving *Clostridia UCG‐014 unclassified*, *Candidatus Saccharimonas*, and *Muribaculaceae unclassified*, they are likely to be involved in the regulation of butyric acid production to improve glycolipid metabolism in T2DM rats. Isovaleric and isobutyric acids are used as by‐products of protein fermentation, especially in microbial protein fermentation (Williams et al. 2016). In our study, we found that the increase in isobutyric acid may be related to LBP regulation of microbial protein fermentation processes (Ma et al. 2022). And involving the *Lachnospiraceae NK4A136 group*, *Monoglobus* and *Desulfovibrionaceae unclassified* are likely to be involved in regulating isobutyric acid production to suppress insulin resistance and improve lipid metabolism. These results suggest that remodeling the composition of gut microbiota is a key strategy to reduce the risk of developing diabetes.

### 4.3 Effects of Exercise and Combined LBP on T2DM

Exercise has several health benefits, including a lower risk of chronic diseases caused by intestinal permeability (Keirns et al. 2020). Exercise, for example, can enhance body composition, reduce weight gain, and mitigate the decline in microbial diversity caused by a high‐fat diet (Campbell et al. 2016). However, the effects of exercise are not limited to increasing microbial diversity (Campbell and Wisniewski 2017). Exercise has been shown to be an effective modulator of SCFAs composition, with specific effects on short‐chain fatty acid salt concentrations. It has been shown that exercise affects the composition of the gut microbiota, increasing SCFAs production and plasma SCFAs concentration to improve IR in skeletal muscle (Yang et al. 2020). The present study also showed that aerobic exercise greatly enhanced the abundance of gut microbiota, raised the levels of SCFAs (particularly isobutyric acid, valeric acid, and hexanoic acid), and lowered blood glucose levels as well as body weight loss in T2DM rats. Additionally, exercise is regarded as an effective non‐pharmacological therapy for reducing inflammatory signaling pathways (Aragón‐Vela et al. 2021). For example, exercise diminishes inflammatory mediators, enhances antioxidant enzymes, and lowers TNF‐α expression in intestinal lymphocytes, while also promoting immune surveillance, thereby exerting an anti‐inflammatory effect on the gut (Campbell and Wisniewski 2017; Rojas‐Valverde et al. 2023). And we proved it in this study.

Further investigation into the gut microbiota's role in exercise is needed to inform personalized interventions for improving glucose‐lipid metabolism and insulin sensitivity, with SCFAs identified as key mediators in these processes (Ticinesi et al. 2019). Our study explored the correlations between gut bacteria, SCFAs, and exercise responses, revealing that aerobic exercise and LBP exerted generally consistent effects on the gut microbiota—though with subtle differences in SCFA modulation. Notably, the *Lachnospiraceae NK4A136 group*, *Monoglobus*, *Desulfovibrionaceae unclassified*, *Phascolarctobacterium*, *Desulfovibrio*, *Enterorhabdus*, and their associated SCFAs (hexanoic and isobutyric acids) likely play critical roles in aerobic exercise‐induced T2DM improvement. In addition, it has been shown that isobutyric acid can regulate energy metabolic processes in the human body through certain signaling pathways (Nogal et al. 2021). This is supported by the finding in our study that gut microbiota involving isobutyric acid was also significantly correlated with HDL‐C and TG/HDL‐C (which indirectly reflects insulin resistance). Additionally, research indicates that alterations in microbiota due to exercise can mitigate the clinical manifestations of inflammatory bowel disease (Bilski et al. 2016). This study found that the increased abundances of *Intestinimonas* and *Colidextribacter* and the decreased abundance of *Kineothrix* significantly improved the inflammatory response (reducing the levels of IL‐6 and TNF‐α). In contrast, SCFAs involved in the effects of aerobic exercise were not found to be involved in the regulation of the inflammatory response, suggesting that the three genera mentioned above may influence the inflammatory process in T2DM rats in other ways.

Building on prior studies of aerobic exercise or LBP alone, our study added a combined intervention group to elucidate the synergistic mechanisms of exercise and LBP in T2DM. Results showed that combined intervention significantly upregulated isovaleric, isobutyric, butyric, and hexanoic acids in T2DM rats, accompanied by activation of functionally relevant gut microbiota (e.g., *Lachnospiraceae NK4A136 group*, *Clostridia UCG‐014*, *Monoglobus*, *Phascolarctobacterium*, *Intestinimonas*, *Muribaculaceae*, *Enterorhabdus*, *Candidatus Saccharimonas*, and *Desulfovibrionaceae unclassified*). Compared to single interventions, aerobic exercise combined with LBP exerted more pronounced effects on regulating glucose‐lipid metabolism, reducing inflammation, and improving IR—providing new theoretical and practical insights for the comprehensive prevention and treatment of T2DM.

### 4.4 Effect of Exercise and Combined LBP on Molecular Pathways in Type 2 Diabetes Through Gut Microbiota

AMPK serves as a crucial regulator of lipid and carbohydrate metabolism. Its activation promotes ATP‐generating pathways, including glucose transport and fatty acid oxidation (Cantó and Auwerx 2009; Huang et al. 2019). A recent study found that AMPK activation reduced cardiac apoptosis in diabetic mice (Xue et al. 2022). Furthermore, SCFAs can directly activate AMPK by increasing the AMP/ATP ratio, which regulates the activity of lipid metabolism‐related proteins and cholesterol‐glucose levels in muscles (Wegierska et al. 2022). As a downstream molecule of the AMPK pathway, PGC‐1α is involved in regulating mitochondrial gene synthesis and plays a key role in glucose homeostasis regulation (Wang et al. 2020). Moreover, the dysregulation of oxidative stress and apoptosis mediated by PGC‐1α is linked to diabetes and its complications. Decreased levels of PGC‐1α have been noted in multiple kidney disease models, while activated PGC‐1α has been shown to mitigate oxidative damage and apoptosis in vitro (Liao et al. 2019). In this study, we studied the relative expression levels of the AMPK/PGC‐1α pathway. We discovered that the activity of this system was down‐regulated and the degree of oxidative stress was heightened in T2DM rats (decreased SOD activity and increased MDA content). Furthermore, aerobic exercise and LBP may affect the expression of AMPK/PGC‐1α pathway by regulating *Firmicutes unclassified* (increased abundance) and *Kineothrix* (decreased abundance), thereby improving oxidative stress and promoting lipid metabolism.

Additionally, studies have shown that butyric acid can effectively alleviate inflammatory responses and insulin resistance and play an important role in intestinal barrier function (Knudsen et al. 2018). Furthermore, butyrate has the ability to affect mitochondrial function. Butyrate has been shown to alleviate mitochondrial dysfunction and kidney injury in rats with diabetic nephropathy through the AMPK/PGC‐1α pathway (Lu et al. 2024). In our study, we also found that aerobic exercise combined with LBP intervention increased butyric acid content in the bodies of T2DM rats. Moreover, *Clostridia UCG‐014 unclassified*, *Muribaculaceae unclassified*, and *Candidatus Saccharimonas* were significantly associated with butyric acid, suggesting that the above genera are likely to be involved in butyric acid synthesis in the context of aerobic exercise combined with the intervention of LBP and, by doing so, influence the expression of the AMPK/PGC‐1α pathway to improve the disease in T2DM rats. It is suggested that the above genera may be involved in butyric acid synthesis in the context of aerobic exercise combined with LBP intervention and, through this, affect the expression of the AMPK/PGC‐1α pathway to improve the disease in T2DM rats.

## 5 Conclusion

Aerobic exercise and LBP can improve insulin resistance caused by T2DM, promote the proliferation of beneficial bacteria, reduce harmful bacteria, optimize the intestinal microecology, and then enhance the sensitivity of body cells to insulin, helping to control sugar smoothly. At the same time, aerobic exercise and LBP can regulate the composition and metabolism of gut microbiota and effectively inhibit inflammatory response. At the level of glucose and lipid metabolism, the two can optimize the process of sugar decomposition and metabolism, regulate lipid metabolism, reduce the abnormal accumulation of blood lipids, and regulate the metabolic imbalance in all aspects of the body, which is helpful for the improvement of the condition of T2DM by improving the environment of gut microbiota. Aerobic exercise combined with LBP has a more significant effect on the regulation of gut microbiota, which has a better effect on the improvement of T2DM.

In addition, the molecular mechanisms by which aerobic exercise and LBP regulate the gut microbiota of T2DM rats both involve AMPK/PGC‐1α regulation, which may be a key pathway between gut microbiota and T2DM. Aerobic exercise and LBP may affect the expression of AMPK/PGC‐1α pathway by regulating *Firmicutes unclassified* and *Kineothrix*, thereby improving oxidative stress and promoting lipid metabolism. Moreover, *Clostridia UCG‐014 unclassified*, *Muribaculaceae unclassified*, and *Candidatus Saccharimona* are likely to be involved in butyric acid synthesis under the intervention of aerobic exercise in combination with LBP, and in this way affect the expression of the AMPK/PGC‐1α pathway to play a role in ameliorating inflammation in T2DM rats. In addition, isobutyric acid and the gut microbiota associated with it may play a key role in the improvement of T2DM with aerobic exercise and LBP, which could be focused on in the future.

## Author Contributions

Conceptualization: J.W. Data curation: J.W., X.C. Formal analysis: J.W., X.L. Funding acquisition: X.L. Investigation: S.F. Methodology: J.W., S.F., X.C, X.L. Project administration: X.L. Resources: X.L. Software: J.W., S.F., X.C. Supervision: X.L. Validation: S.F., X.L. Visualization: J.W., S.F., X.C. Writing – original draft: J.W. Writing – review and editing: J.W., X.L.

## Funding

This work is supported by the Natural Science Foundation of Heilongjiang Province (LH2024G003) and Harbin sport university Intra‐school Doctoral Talent Research Initiation Fund Project (RCYJ‐2105).

## Conflicts of Interest

The authors declare no conflicts of interest.

## Supporting information


**Table S1:** Experimental reagents.

**Table S2:** Experimental equipment.

## Data Availability

Data is provided within the manuscript.

## References

[fsn371503-bib-0001] Aragón‐Vela, J. , P. Solis‐Urra , F. Ruiz‐Ojeda , A. Alvarez‐Mercado , J. Olivares‐Arancibia , and J. Plaza‐Diaz . 2021. “Impact of Exercise on Gut Microbiota in Obesity.” Nutrients 13, no. 11: 3999. 34836254 10.3390/nu13113999 PMC8624603

[fsn371503-bib-0002] Balducci, S. , M. Sacchetti , J. Haxhi , et al. 2014. “Physical Exercise as Therapy for Type 2 Diabetes Mellitus.” Diabetes/Metabolism Research and Reviews 30: 13–23. 24353273 10.1002/dmrr.2514

[fsn371503-bib-0003] Bäumler, A. , and V. Sperandio . 2016. “Interactions Between the Microbiota and Pathogenic Bacteria in the Gut.” Nature 535: 85–93. 27383983 10.1038/nature18849 PMC5114849

[fsn371503-bib-0004] Bilski, J. , A. MAZUR‐Bialy , B. Brzozowski , et al. 2016. “Can Exercise Affect the Course of Inflammatory Bowel Disease? Experimental and Clinical Evidence.” Pharmacological Reports 68: 827–836. 27255494 10.1016/j.pharep.2016.04.009

[fsn371503-bib-0005] Campbell, S. , and P. Wisniewski . 2017. “Exercise Is a Novel Promoter of Intestinal Health and Microbial Diversity.” Exercise and Sport Sciences Reviews 45: 41–47. 27782912 10.1249/JES.0000000000000096

[fsn371503-bib-0006] Campbell, S. , P. Wisniewski , M. Noji , et al. 2016. “The Effect of Diet and Exercise on Intestinal Integrity and Microbial Diversity in Mice.” PLoS One 11, no. 3: e0150502. 26954359 10.1371/journal.pone.0150502 PMC4783017

[fsn371503-bib-0007] Cantó, C. , and J. Auwerx . 2009. “PGC‐1α, SIRT1 and Ampk, an Energy Sensing Network That Controls Energy Expenditure.” Current Opinion in Lipidology 20: 98–105. 19276888 10.1097/MOL.0b013e328328d0a4 PMC3627054

[fsn371503-bib-0008] Cao, C. , L. Wang , C. Ai , et al. 2022. “Impact of *Lycium barbarum* Arabinogalactan on the Fecal Metabolome in a DSS‐Induced Chronic Colitis Mouse Model.” Food & Function 13: 8703–8716. 35912853 10.1039/d2fo01283a

[fsn371503-bib-0009] Cao, C. , Z. Wang , G. Gong , et al. 2022. “Effects of *Lycium barbarum* Polysaccharides on Immunity and Metabolic Syndrome Associated With the Modulation of Gut Microbiota: A Review.” Food 11, no. 20: 3177. 10.3390/foods11203177 PMC9602392 37430929

[fsn371503-bib-0010] Chiasera, J. , K. WARD‐Cook , S. Mccune , and G. Wardlaw . 2000. “Effect of Aerobic Training on Diabetic Nephropathy in a Rat Model of Type 2 Diabetes Mellitus.” Annals of Clinical and Laboratory Science 30: 346–353. 11045758

[fsn371503-bib-0011] de Vadder, F. , P. Kovatcheva‐Datchary , D. Goncalves , et al. 2014. “Microbiota‐Generated Metabolites Promote Metabolic Benefits via Gut‐Brain Neural Circuits.” Cell 156: 84–96. 24412651 10.1016/j.cell.2013.12.016

[fsn371503-bib-0012] den Besten, G. , K. van Eunen , A. Groen , K. Venema , D. Reijngoud , and B. Bakker . 2013. “The Role of Short‐Chain Fatty Acids in the Interplay Between Diet, Gut Microbiota, and Host Energy Metabolism.” Journal of Lipid Research 54: 2325–2340. 23821742 10.1194/jlr.R036012 PMC3735932

[fsn371503-bib-0013] Ding, H. , P. Yang , X. Zhang , and Y. Ma . 2022. “Efficacy of Pretreatment With *Lycium Barbarum* Polysaccharide in Various Doses in Influencing Splenic Immunity and Prognosis of Sepsis in Rats.” Evidence‐based Complementary and Alternative Medicine 2022: 1–13. 10.1155/2022/9508603 PMC9553460 36248408

[fsn371503-bib-0014] Frank, J. , A. Gupta , V. Osadchiy , and E. Mayer . 2021. “Brain‐Gut‐Microbiome Interactions and Intermittent Fasting in Obesity.” Nutrients 13, no. 2: 584. 33578763 10.3390/nu13020584 PMC7916460

[fsn371503-bib-0015] Guo, Q. , Y. Li , X. Dai , B. Wang , J. Zhang , and H. Cao . 2023. “Polysaccharides: The Potential Prebiotics for Metabolic Associated Fatty Liver Disease (MAFLD).” Nutrients 15, no. 17: 3722. 37686754 10.3390/nu15173722 PMC10489936

[fsn371503-bib-0016] Han, Q. , J. Wang , W. Li , Z. Chen , and Y. Du . 2021. “Androgen‐Induced Gut Dysbiosis Disrupts Glucolipid Metabolism and Endocrinal Functions in Polycystic Ovary Syndrome.” Microbiome 9, no. 1: 101. 33957990 10.1186/s40168-021-01046-5 PMC8103748

[fsn371503-bib-0017] Haram, P. , O. Kemi , S. Lee , et al. 2009. “Aerobic Interval Training vs. Continuous Moderate Exercise in the Metabolic Syndrome of Rats Artificially Selected for Low Aerobic Capacity.” Cardiovascular Research 81: 723–732. 19047339 10.1093/cvr/cvn332 PMC2642601

[fsn371503-bib-0018] He, J. , P. Zhang , L. Shen , et al. 2020. “Short‐Chain Fatty Acids and Their Association With Signalling Pathways in Inflammation, Glucose and Lipid Metabolism.” International Journal of Molecular Sciences 21, no. 17: 6356. 32887215 10.3390/ijms21176356 PMC7503625

[fsn371503-bib-0019] Henao‐Mejia, J. , E. Elinav , C. Jin , et al. 2012. “Inflammasome‐Mediated Dysbiosis Regulates Progression of NAFLD and Obesity.” Nature 482: 179. 22297845 10.1038/nature10809 PMC3276682

[fsn371503-bib-0020] Huang, H. , A. Aminian , M. Hassan , et al. 2019. “Gastric Bypass Surgery Improves the Skeletal Muscle Ceramide/S1P Ratio and Upregulates the AMPK/ SIRT1/ PGC‐1α Pathway in Zucker Diabetic Fatty Rats.” Obesity Surgery 29: 2158–2165. 30809769 10.1007/s11695-019-03800-z

[fsn371503-bib-0021] Iatcu, C. , A. Steen , and M. Covasa . 2022. “Gut Microbiota and Complications of Type‐2 Diabetes.” Nutrients 14, no. 1: 166. 10.3390/nu14010166 PMC8747253 35011044

[fsn371503-bib-0022] Kang, N. , G. Chen , R. Tu , et al. 2022. “Adverse Associations of Different Obesity Measures and the Interactions With Long‐Term Exposure to Air Pollutants With Prevalent Type 2 Diabetes Mellitus: The Henan Rural Cohort Study.” Environmental Research 207: 112640. 34990613 10.1016/j.envres.2021.112640

[fsn371503-bib-0023] Keirns, B. , N. Koemel , C. Sciarrillo , K. Anderson , and S. Emerson . 2020. “Exercise and Intestinal Permeability: Another Form of Exercise‐Induced Hormesis?” American Journal of Physiology. Gastrointestinal and Liver Physiology 319: G512–G518. 32845171 10.1152/ajpgi.00232.2020

[fsn371503-bib-0024] Kleinert, M. , C. Clemmensen , S. Hofmann , et al. 2018. “Animal Models of Obesity and Diabetes Mellitus.” Nature Reviews Endocrinology 14: 140–162. 10.1038/nrendo.2017.161 29348476

[fsn371503-bib-0025] Knudsen, K. , H. Lærke , M. Hedemann , et al. 2018. “Impact of Diet‐Modulated Butyrate Production on Intestinal Barrier Function and Inflammation.” Nutrients 10, no. 10: 1499. 30322146 10.3390/nu10101499 PMC6213552

[fsn371503-bib-0026] Koh, A. , F. DE Vadder , P. KOVATCHEVA‐Datchary , and F. Bäckhed . 2016. “From Dietary Fiber to Host Physiology: Short‐Chain Fatty Acids as Key Bacterial Metabolites.” Cell 165: 1332–1345. 27259147 10.1016/j.cell.2016.05.041

[fsn371503-bib-0027] La Fata, G. , P. Weber , and M. Mohajeri . 2018. “Probiotics and the Gut Immune System: Indirect Regulation.” Probiotics and Antimicrobial Proteins 10: 11–21. 28861741 10.1007/s12602-017-9322-6 PMC5801397

[fsn371503-bib-0028] Lai, W. , C. Wang , R. Lai , X. Peng , and J. Luo . 2022. “ *Lycium Barbarum* Polysaccharide Modulates Gut Microbiota to Alleviate Rheumatoid Arthritis in a Rat Model.” npj Science of Food 6: 34. 35864275 10.1038/s41538-022-00149-z PMC9304368

[fsn371503-bib-0029] Li, S. , and Y. Guo . 2023. “Gut Microbiome: New Perspectives for Type 2 Diabetes Prevention and Treatment.” World Journal of Clinical Cases 11: 7508–7520. 38078135 10.12998/wjcc.v11.i31.7508 PMC10698456

[fsn371503-bib-0030] Li, Z. , J. Zhuo , N. Yang , Z. Qu , and T. Han . 2024. “Effect of *Lycium barbarum* Polysaccharide on Osteoblast Proliferation and Differentiation in Postmenopausal Osteoporosis.” International Journal of Biological Macromolecules 271, no. 1: 132415. 38759858 10.1016/j.ijbiomac.2024.132415

[fsn371503-bib-0031] Liao, Z. , J. Zhang , J. Wang , et al. 2019. “The Anti‐Nephritic Activity of a Polysaccharide From Okra (*Abelmoschus esculentus* (L.) Moench) via Modulation of AMPK‐Sirtl‐PGC‐1α Signaling Axis Mediated Anti‐Oxidative in Type 2 Diabetes Model Mice.” International Journal of Biological Macromolecules 140: 568–576. 31442509 10.1016/j.ijbiomac.2019.08.149

[fsn371503-bib-0032] Lin, H. , A. Frassetto , E. Kowalik , et al. 2012. “Butyrate and Propionate Protect Against Diet‐Induced Obesity and Regulate Gut Hormones via Free Fatty Acid Receptor 3‐Independent Mechanisms.” PLoS One 7, no. 4: e35240. 22506074 10.1371/journal.pone.0035240 PMC3323649

[fsn371503-bib-0033] Lin, S. , S. Jin , F. Zhou , Y. Hu , and M. Zhang . 2021. “Effects of Endurance Exercise on Serum Inflammatory Cytokine Level and Kidney Structure in a Rat Diabetes Model.” Experimental and Therapeutic Medicine 22, no. 4: 1125. 34466141 10.3892/etm.2021.10559 PMC8383327

[fsn371503-bib-0034] Liu, L. , J. Zhang , Y. Cheng , et al. 2022. “Gut Microbiota: A New Target for T2DM Prevention and Treatment.” Frontiers in Endocrinology 13: 958218. 36034447 10.3389/fendo.2022.958218 PMC9402911

[fsn371503-bib-0035] Liu, Q. , Q. Han , M. Lu , H. Wang , and F. Tang . 2019. “ *Lycium barbarum* Polysaccharide Attenuates Cardiac Hypertrophy, Inhibits Calpain‐1 Expression and Inhibits NF‐κB Activation in Streptozotocin‐Induced Diabetic Rats.” Experimental and Therapeutic Medicine 18: 509–516. 31258688 10.3892/etm.2019.7612 PMC6566019

[fsn371503-bib-0036] Liu, R. , Y. He , H. Liu , D. Zheng , S. Huang , and C. Liu . 2021. “Protective Effect of *Lycium barbarum* Polysaccharide on di‐(2‐Ethylhexyl) Phthalate‐Induced Toxicity in Rat Liver.” Environmental Science and Pollution Research 28: 23501–23509. 33449321 10.1007/s11356-020-11990-8

[fsn371503-bib-0037] Lu, L. , L. Liu , L. Li , Y. Hu , X. Xian , and W. Li . 2024. “Sodium Butyrate Improves Cognitive Dysfunction in High‐Fat Diet/ Streptozotocin‐Induced Type 2 Diabetic Mice by Ameliorating Hippocampal Mitochondrial Damage Through Regulating AMPK/PGC‐1α Pathway.” Neuropharmacology 261: 110139. 39233201 10.1016/j.neuropharm.2024.110139

[fsn371503-bib-0038] Ma, Q. , Y. Li , P. Li , et al. 2019. “Research Progress in the Relationship Between Type 2 Diabetes Mellitus and Intestinal Flora.” Biomedicine & Pharmacotherapy 117: 109138. 31247468 10.1016/j.biopha.2019.109138

[fsn371503-bib-0039] Ma, Q. , R. Zhai , X. Xie , et al. 2022. “Hypoglycemic Effects of *Lycium barbarum* Polysaccharide in Type 2 Diabetes Mellitus Mice *via* Modulating Gut Microbiota.” Frontiers in Nutrition 9: 916271. 35845787 10.3389/fnut.2022.916271 PMC9280299

[fsn371503-bib-0040] Mokkala, K. , N. Houttu , T. Cansev , and K. Laitinen . 2020. “Interactions of Dietary Fat With the Gut Microbiota: Evaluation of Mechanisms and Metabolic Consequences.” Clinical Nutrition 39: 994–1018. 31171289 10.1016/j.clnu.2019.05.003

[fsn371503-bib-0041] Motiani, K. , M. Collado , J. Eskelinen , et al. 2020. “Exercise Training Modulates Gut Microbiota Profile and Improves Endotoxemia.” Medicine & Science in Sports & Exercise 52: 94–104. 31425383 10.1249/MSS.0000000000002112 PMC7028471

[fsn371503-bib-0042] Nogal, A. , A. Valdes , and C. Menni . 2021. “The Role of Short‐Chain Fatty Acids in the Interplay Between Gut Microbiota and Diet in Cardio‐Metabolic Health.” Gut Microbes 13, no. 1: 1–24. 10.1080/19490976.2021.1897212 PMC8007165 33764858

[fsn371503-bib-0043] Palmas, V. , S. Pisanu , V. Madau , et al. 2021. “Gut Microbiota Markers Associated With Obesity and Overweight in Italian Adults.” Scientific Reports 11, no. 1: 5532. 33750881 10.1038/s41598-021-84928-w PMC7943584

[fsn371503-bib-0044] Pedersen, H. , V. Gudmundsdottir , H. Nielsen , et al. 2016. “Human Gut Microbes Impact Host Serum Metabolome and Insulin Sensitivity.” Nature 535: 376. 27409811 10.1038/nature18646

[fsn371503-bib-0045] Peng, J. , X. Li , L. Zheng , et al. 2022. “Ban‐Lan‐Gen Granule Alleviates Dextran Sulfate Sodium‐Induced Chronic Relapsing Colitis in Mice via Regulating Gut Microbiota and Restoring Gut SCFA Derived‐GLP‐1 Production.” Journal of Inflammation Research 15: 1457–1470. 35250294 10.2147/JIR.S352863 PMC8896204

[fsn371503-bib-0046] Perdijk, O. , R. Azzoni , and B. Marsland . 2024. “The Microbiome: An Integral Player in Immune Homeostasis and Inflammation in the Respiratory Tract.” Physiological Reviews 104: 835–879. 38059886 10.1152/physrev.00020.2023

[fsn371503-bib-0047] Pi, Y. , M. Fang , Y. Li , et al. 2024. “Interactions Between Gut Microbiota and Natural Bioactive Polysaccharides in Metabolic Diseases: Review.” Nutrients 16, no. 17: 2838. 39275156 10.3390/nu16172838 PMC11397228

[fsn371503-bib-0048] Ratajczak, W. , A. Ryl , A. Mizerski , K. Walczakiewicz , O. Sipak , and M. Laszczynska . 2019. “Immunomodulatory Potential of Gut Microbiome‐Derived Short‐Chain Fatty Acids (SCFAs).” Acta Biochimica Polonica 66: 1–12. 30831575 10.18388/abp.2018_2648

[fsn371503-bib-0049] Ren, F. , C. Meng , W. Chen , H. Chen , and W. Chen . 2021. “Ganoderma Amboinense Polysaccharide Prevents Obesity by Regulating Gut Microbiota in High‐Fat‐Diet Mice.” Food Bioscience 42: 101107.

[fsn371503-bib-0050] Rojas‐Valverde, D. , D. Bonilla , L. Gómez‐Miranda , J. Calleja‐Núñez , N. Arias , and I. Martínez‐Guardado . 2023. “Examining the Interaction Between Exercise, Gut Microbiota, and Neurodegeneration: Future Research Directions.” Biomedicine 11, no. 8: 2267. 10.3390/biomedicines11082267 PMC10452292 37626763

[fsn371503-bib-0051] Saraceni, C. , and T. Broderick . 2007. “Cardiac and Metabolic Consequences of Aerobic Exercise Training in Experimental Diabetes.” Current Diabetes Reviews 3: 75–84. 18220658 10.2174/157339907779802111

[fsn371503-bib-0052] Sawicki, C. , K. Livingston , M. Obin , S. Roberts , M. Chung , and N. Mckeown . 2017. “Dietary Fiber and the Human Gut Microbiota: Application of Evidence Mapping Methodology.” Nutrients 9, no. 2: 125. 28208609 10.3390/nu9020125 PMC5331556

[fsn371503-bib-0053] Sowmiya, T. , and S. Silambanan . 2023. “Association of Gut Microbiota and Diabetes Mellitus.” Current Diabetes Reviews 19, no. 7: e211122211066. 36411560 10.2174/1573399819666221121104542

[fsn371503-bib-0054] Tanca, A. , M. Abbondio , A. Palomba , et al. 2017. “Potential and Active Functions in the Gut Microbiota of a Healthy Human Cohort.” Microbiome 5, no. 1: 79. 28709472 10.1186/s40168-017-0293-3 PMC5513205

[fsn371503-bib-0055] Ticinesi, A. , F. Lauretani , C. Tana , A. Nouvenne , E. Ridolo , and T. Nleschi . 2019. “Exercise and Immune System as Modulators of Intestinal Microbiome: Implications for the Gut‐Muscle Axis Hypothesis.” Exercise Immunology Review 25: 8–19. 30753131

[fsn371503-bib-0056] Wang, S. , S. Zhu , J. Wu , et al. 2020. “Exercise Enhances Cardiac Function by Improving Mitochondrial Dysfunction and Maintaining Energy Homoeostasis in the Development of Diabetic Cardiomyopathy.” Journal of Molecular Medicine 98: 245–261. 31897508 10.1007/s00109-019-01861-2

[fsn371503-bib-0057] Wang, X. , X. Wang , H. Jiang , et al. 2018. “Marine Polysaccharides Attenuate Metabolic Syndrome by Fermentation Products and Altering Gut Microbiota: An Overview.” Carbohydrate Polymers 195: 601–612. 29805017 10.1016/j.carbpol.2018.05.003

[fsn371503-bib-0058] Wegierska, A. , I. Charitos , S. Topi , M. Potenza , M. Montagnani , and L. Santacroce . 2022. “The Connection Between Physical Exercise and Gut Microbiota: Implications for Competitive Sports Athletes.” Sports Medicine 52: 2355–2369. 35596883 10.1007/s40279-022-01696-x PMC9474385

[fsn371503-bib-0059] Williams, B. , D. Zhang , A. Lisle , et al. 2016. “Soluble Arabinoxylan Enhances Large Intestinal Microbial Health Biomarkers in Pigs Fed a Red Meat‐Containing Diet.” Nutrition 32: 491–497. 26740253 10.1016/j.nut.2015.10.008

[fsn371503-bib-0060] Xue, F. , J. Cheng , Y. Liu , et al. 2022. “Cardiomyocyte‐Specific Knockout of ADAM17 Ameliorates Left Ventricular Remodeling and Function in Diabetic Cardiomyopathy of Mice.” Signal Transduction and Targeted Therapy 7: 259. 35909160 10.1038/s41392-022-01054-3 PMC9339545

[fsn371503-bib-0061] Yang, L. , H. Lin , W. Lin , and X. Xu . 2020. “Exercise Ameliorates Insulin Resistance of Type 2 Diabetes Through Motivating Short‐Chain Fatty Acid‐Mediated Skeletal Muscle Cell Autophagy.” Biology 9, no. 8: 203. 32756447 10.3390/biology9080203 PMC7464264

[fsn371503-bib-0062] Yehualashet, A. , and B. Yikna . 2021. “Microbial Ecosystem in Diabetes Mellitus: Consideration of the Gastrointestinal System.” Diabetes Metabolic Syndrome and Obesity‐Targets and Therapy 14: 1841–1854. 33953584 10.2147/DMSO.S304497 PMC8089103

[fsn371503-bib-0063] Yi, C. , S. Huang , W. Zhang , et al. 2025. “Synergistic Interactions Between Gut Microbiota and Short Chain Fatty Acids: Pioneering Therapeutic Frontiers in Chronic Disease Management.” Microbial Pathogenesis 199: 107231. 39681288 10.1016/j.micpath.2024.107231

[fsn371503-bib-0064] Zhang, L. , H. Lin , X. Yang , et al. 2023. “Effects of Dapagliflozin Monotherapy and Combined Aerobic Exercise on Skeletal Muscle Mitochondrial Quality Control and Insulin Resistance in Type 2 Diabetes Mellitus Rats.” Biomedicine & Pharmacotherapy 169: 115852. 37944441 10.1016/j.biopha.2023.115852

[fsn371503-bib-0065] Zhang, X. , N. Zhang , J. Kan , et al. 2020. “Anti‐Inflammatory Activity of Alkali‐Soluble Polysaccharides From *Arctium lappa* L. and Its Effect on Gut Microbiota of Mice With Inflammation.” International Journal of Biological Macromolecules 154: 773–787. 32199919 10.1016/j.ijbiomac.2020.03.111

[fsn371503-bib-0066] Zheng, Y. , S. Ley , and F. Hu . 2018. “Global Aetiology and Epidemiology of Type 2 Diabetes Mellitus and Its Complications.” Nature Reviews Endocrinology 14: 88–98. 10.1038/nrendo.2017.151 29219149

[fsn371503-bib-0067] Zhou, Z. , B. Sun , D. Yu , and C. Zhu . 2022. “Gut Microbiota: An Important Player in Type 2 Diabetes Mellitus.” Frontiers in Cellular and Infection Microbiology 12: 834485. 35242721 10.3389/fcimb.2022.834485 PMC8886906

[fsn371503-bib-0068] Zhu, W. , S. Zhou , J. Liu , R. Mclean , and W. Chu . 2020. “Prebiotic, Immuno‐Stimulating and Gut Microbiota‐Modulating Effects of *Lycium barbarum* Polysaccharide.” Biomedicine & Pharmacotherapy 121: 109591. 31733576 10.1016/j.biopha.2019.109591

