# Supplementary material for: Ameliorative Effects of Aerobic Exercise Combined With Lycium barbarum Polysaccharide‐Mediated Gut Microbiota Remodeling on Glycolipid Abnormalities in Type 2 Diabetic Rats
Source: Food Sci Nutr. 2026 Feb 1;14(2):e71503. doi: 10.1002/fsn3.71503 (PMC12862017; doi:10.1002/fsn3.71503)
Supplement: Supplementary file 1 — Table S1: Experimental reagents. [file FSN3-14-e71503-s002.docx]

**Supplementary Table 1. Experimental reagents.**

| **Name of reagent** | **Manufacturers** |
| --- | --- |
| Streptozotocin (STZ) | Sigma-Aldrich |
| Sodium Citrate Buffer | Solarbio |
| Sodium Pentobarbital | Sigma-Aldrich |
| 0.9% Sodium Chloride Injection | Harbin Sanlian Pharmaceutical Co., Ltd. |
| Rat Total Cholesterol (TCHO) Assay Kit | Nanjing Jiancheng Institute of Bioengineering |
| Rat Triglyceride (TG) Assay Kit | Nanjing Jiancheng Institute of Bioengineering |
| Rat Low-Density Lipoprotein Cholesterol (LDL-C) Assay Kit | Nanjing Jiancheng Institute of Bioengineering |
| Rat High-Density Lipoprotein Cholesterol (HDL-C) Assay Kit | Nanjing Jiancheng Institute of Bioengineering |
| Rat Insulin (INS) Assay Kit | Jiangsu Meimian Industry Co., Ltd. |
| Rat Glucagon-Like Peptide 1 (GLP-1) Assay Kit | Jianglai Biotechnology Co., Ltd. |
| Rat Interleukin-6 (IL-6) Assay Kit | R&D Systems |
| Rat Tumor Necrosis Factor-α (TNF-α) Assay Kit | R&D Systems |
| Rat Superoxide Dismutase (SOD) Assay Kit | Cayman Chemical |
| Rat Malondialdehyde (MDA) Assay Kit | Cayman Chemical |
| Rat Adenosine Monophosphate-Activated Protein Kinase (AMPK) Assay Kit | Abcam |
| Rat Peroxisome Proliferator-Activated Receptor Gamma Coactivator 1-Alpha (PGC-1α) Assay Kit | Abcam |
| Lycium barbarum Polysaccharide | Shanghai Yuanye Biotechnology Co., Ltd. |
