# Supplementary material for: Ameliorative Effects of Aerobic Exercise Combined With Lycium barbarum Polysaccharide‐Mediated Gut Microbiota Remodeling on Glycolipid Abnormalities in Type 2 Diabetic Rats
Source: Food Sci Nutr. 2026 Feb 1;14(2):e71503. doi: 10.1002/fsn3.71503 (PMC12862017; doi:10.1002/fsn3.71503)
Supplement: Supplementary file 2 — Table S2: Experimental equipment. [file FSN3-14-e71503-s001.docx]

**Supplementary Table 2. Experimental equipment.**

| **Equipment name** | **Manufacturers** |
| --- | --- |
| -80℃ Ultra-Low Temperature Freezer | Thermo Fisher Scientific Inc. (USA) |
| Electronic Balance | Mettler-Toledo International Inc. (Switzerland) |
| Precision Electronic Balance | Sartorius AG (Germany) |
| Bench-top High-Speed Refrigerated Centrifuge | Beijing DLAB Scientific Co., Ltd. |
| Laboratory Ultra-Pure Water System | Aiken Water Genie |
| ELISA Reader | BioTek Instruments Inc. (USA) |
| Real-Time Fluorescent Quantitative PCR | Bioer Technology Co., Ltd. |
| Animal Exercise Treadmill | Anhui Zhenghua Biological Instruments Co., Ltd. |
| Portable Blood Glucose Meter | Aike Biotechnology Co., Ltd. |
| Pipette | Eppendorf Life Sciences GmbH (Germany) |
| Incubator | Shanghai Shangdao Instrument Manufacturing Co., Ltd. |
| Bench-Top Centrifuge | Shanghai Anting Co., Ltd. |
| Gavage Needle | Zhongke Life Science Technology Co., Ltd. |
